# The impact of cuts in the US President’s Emergency Plan for AIDS Relief funding for HIV pre-exposure prophylaxis in sub-Saharan Africa: a modelling study

**DOI:** 10.1016/S2352-3018(25)00192-4

**Published:** 2025-10-01

**Authors:** Jack Stone, Kennedy Kipkoech Mutai, Adelina Artenie, Romain Silhol, Marie-Claude Boily, Jirair Ratevosian, Chris Beyrer, Peter Vickerman

**Affiliations:** Population Health Sciences, Bristol Medical School, University of Bristol, Bristol, UK; Population Health Sciences, Bristol Medical School, University of Bristol, Bristol, UK; University of Montreal, Montreal, QC, Canada; Medical Research Council Centre for Global Infectious Disease Analysis, School of Public Health, Imperial College London, London, UK; HIV Prevention Trials Network Modelling Centre, Imperial College London, London, UK; Duke Global Health Institute, Duke University, Durham, NC, USA; Population Health Sciences, Bristol Medical School, University of Bristol, Bristol, UK

## Abstract

**Background:**

In January, 2025, the US Government issued a directive, pausing all foreign aid programmes. This directive included a 90-day pausing of all US President’s Emergency Plan for AIDS Relief (PEPFAR) funding for HIV oral pre-exposure prophylaxis (PrEP) except for pregnant and breastfeeding women, with a return to funding for PrEP looking increasingly unlikely. We aimed to estimate the impact of a funding pause for PrEP on HIV infections in sub-Saharan Africa.

**Methods:**

In this mathematical modelling study, we developed a static HIV transmission model incorporating PrEP, parameterised with estimates of population size, HIV prevalence and incidence, and PrEP effectiveness for different subpopulations (including key populations) in each PEPFAR-funded sub-Saharan African country. Key populations were men who have sex with men, female sex workers, transgender women, and people who inject drugs. We used PEPFAR reporting on numbers of people in different subpopulations returning for oral PrEP for each country in July to September, 2024, as the estimated number using oral PrEP provided by PEPFAR. For each country and subpopulation, we modelled the relative and absolute increase in new primary HIV infections resulting from removing this funded PrEP for a year and the number of secondary infections that could result from these primary infections during the next 5 years.

**Findings:**

Towards the end of 2024, 719 384 individuals who were not breastfeeding or pregnant, including 205 868 people from key populations, received PEPFAR-funded PrEP across 28 sub-Saharan African countries. The estimated proportion of HIV-negative key population individuals receiving PEPFAR-funded PrEP (ie, the coverage) ranged from 2·6% (95% uncertainty interval 2·0–3·4) in people who inject drugs to 5·0% (4·5–5·9) in female sex workers. Estimated coverage among non-key population men was less than 0·1% (<0·1 to <0·1) and in women was 0·1% (0·1 to 0·1). Stopping PEPFAR’s provision of PrEP for a year could lead to 6671 (5032–8192) additional new primary HIV infections, including 5663 (4146–7074) in key populations. Within key populations, this absolute increase corresponds to relative increases in new HIV infections of 0·8% (0·3–1·5) in people who inject drugs, 1·4% (0·8–2·3) in transgender women, 2·2% (1·3–3·0) in men who have sex with men, and 2·9% (1·8–4·4) in female sex workers. In Zambia, the country with the highest PEPFAR coverage across all key populations, this increase ranges from 7·8% (2·5–17·1) in people who inject drugs to 18·1% (9·7–33·2) in men who have sex with men. When considering secondary transmissions, we estimated that a 1-year pause could lead to 10 313 (7796–12 921) additional infections during the next 5 years.

**Interpretation:**

Our projections show the substantial detrimental impacts that cuts to PEPFAR funding could have. Mitigation measures, including funding through alternative international donors or domestic government budgets, are needed to maintain and increase existing coverage levels of PrEP and therefore prevent the detrimental impact of PEPFAR’s funding cuts, particularly in high coverage countries.

**Funding:**

Wellcome Trust.

## Introduction

Since 2003, the US President’s Emergency Plan for AIDS Relief (PEPFAR) has been providing invaluable care, treatment, and prevention services for HIV,^1^ with services currently in 55 countries^1^ and involving a total investment of more than US$120 billion since 2003.^2^ Since its inception, a chief mission of PEPFAR has been to scale up the provision of HIV treatment, with the programme reaching 20·6 million people by September, 2024.^1^ PEPFAR is also an important provider of HIV prevention services, accounting for more than 90% of global initiations on to oral HIV pre-exposure prophylaxis (PrEP) and providing 35 million voluntary medical male circumcisions since 2007.^1^ It has been estimated that PEPFAR has saved 26 million lives,^1^ with PEPFAR focus countries having 52% fewer infections in 2023 than in 2010, compared with a 39% decrease globally.^3^

Importantly, PEPFAR (which is the primary source of global funding for key population programming) has been providing oral PrEP for key populations since 2016, with 661 497 PrEP initiations among female sex workers, men who have sex with men, people who inject drugs, and transgender women in the first three quarters of 2024 and 254 187 returning for PrEP in the third quarter of that year, making up more than a quarter of PEPFAR’s total PrEP provision (PEPFAR, personal communication). Additionally, there were 1 887 562 initiations among other non-key population women and men (ie, those who are not people who inject drugs, female sex workers, men who have sex with men, or transgender women), with 560 920 returning for PrEP in the third quarter of that year (PEPFAR, personal communication).

Considering that key populations are estimated to have 3–94 times higher HIV incidence than the general population, with recent estimates suggesting they accounted for 55% of all global HIV infections in 2022,^4^ it is probable that this PrEP provision is having a considerable impact on reducing new infections among these populations. PEPFAR’s provision of PrEP is currently in jeopardy due to the US Government’s recent stop-work order on all foreign aid, including PEPFAR, at the end of January, 2025.^5^ This order initiated a 90-day review of foreign aid.^5^ Although a waiver was put in place for providing life-saving HIV treatment and PrEP for pregnant and breastfeeding women (3–6% of PEPFAR’s total PrEP provision), all other PrEP services were put on pause “until further notice”.^6,7,8^ No waiver was given for PrEP among key populations or other subpopulations, with funding for PrEP services for these populations largely stopped.^7^ Although the US Congress protected PEPFAR from proposed cuts of $400 million in July, 2025, future funding cuts have been proposed and PEPFAR continues to operate under the waiver.^9^ Even though the US Government’s foreign aid review is still ongoing, it is likely that the provision of PrEP for these groups will cease in many settings. Indeed, even if the pause is temporary, emerging data already suggest that many organisations have had to close or lay-off staff,^6^ meaning a full recovery to previous capacities will be challenging in the short term.

To highlight the importance of continuing funding for PrEP, we aimed to predict the number of HIV infections that could occur in sub-Saharan Africa during the next year if PEPFAR’s provision of oral PrEP is ceased for all individuals other than pregnant and breastfeeding women, and what this would mean in terms of relative increases in the number of new HIV infections for different population groups in each country. We aimed to focus our projections primarily on key populations, but we also extend our estimates to other non-key population groups.

## Methods

### Model design

In this mathematical modelling study, we developed a static model to estimate the 1-year impact of disruptions to PEPFAR funding of HIV oral PrEP at the country level. The model was defined for different subpopulations, including each key population.

For a given subpopulation and country, the model first estimates the coverage of PEPFAR-funded PrEP among HIV-negative individuals in that subpopulation (*C*) as:

(1) $$C=100\times \frac{n}{(1-H)\times N}$$

where *H* is the HIV prevalence in the subpopulation, *N* is the population size of the subpopulation, and *n* is the number of people receiving PrEP funded by PEPFAR in that subpopulation, all at the country level.

Second, with the use of estimates of the effectiveness of PrEP against HIV acquisition (*E*), which varies by subpopulation, the model calculates the relative increase (as a percentage) in the number of new primary HIV infections in that subpopulation during a period without PEPFAR-funded PrEP (*R*) as:

(2) $$R=100\times \frac{E\times n}{(1-H)\times N-E\times n}$$

Equation 2 represents the ratio of the number of primary infections averted by PrEP (equivalently, the increase in infections if PrEP is removed) to the total number of infections occurring in the population accounting for current levels of PrEP use. Additionally, applying country-level HIV incidence estimates (*I*, measured per person-year) for each subpopulation, our model estimates the additional number of new primary HIV infections (*A*) that would occur during the next year in each subpopulation without PEPFAR-funded PrEP as:

(3) A=E×n×I

We also estimated the number of secondary infections (*S*) that could result from these primary infections, by applying model-based country-level estimates of HIV transmission rates specific to each subpopulation (*T*, measured per person-year), as:

(4) S=A×T×t

where *t* estimates the additional person-years of HIV transmission that could result from each primary infection that occurs due to ceasing PrEP for 1 year. For our analysis, we assume a time span for *t* of 5 years, assuming that these additional people who are infected are unlikely to become infected otherwise during this period, unless the incidence is very high. However, we assume that the additional primary infections occur on average halfway through the year without PEPFAR-funded PrEP, and therefore remove 0·5 years from the 5 years.

### Model parameterisation

For key populations, we used systematic reviews published in the past 2 years to parameterise country-level HIV prevalence and population size estimates.^10,11^ For people who inject drugs, we used estimates for both parameters from a global systematic review^10^ and a review of studies in sub-Saharan Africa,^11^ preferentially using estimates from the global review. For men who have sex with men, female sex workers, and transgender women, we used country-specific estimates from the same review for sub-Saharan Africa.^11^ For non-key populations (ie, people who do not inject drugs, are not female sex workers, are not men who have sex with men, or are not transgender women), our model was stratified by sex (male or female) and age (15–24 years, 25–34 years, or 35–49 years). We used UN estimates of population size for each sex and age stratification, and UNAIDS estimates of the number of men and women living with HIV aged 15–24 years and 15–49 years. We allocated the number of people living with HIV aged 25–49 years proportionately to the age groups 25–34 years and 35–49 years based on the relative population size of these age groups.

For HIV incidence, we used systematic reviews from the past 2 years, including a global review for people who inject drugs,^12^ a recent study for people who inject drugs in South Africa,^13^ and regional reviews for men who have sex with men^14^ and female sex workers^15^ in sub-Saharan Africa. We used recent (those whose study midpoint was in or after 2015) estimates of HIV incidence for each country from these reviews, pooling multiple estimates when available. For countries with missing incidence estimates for people who inject drugs (13 of 15), we used the published pooled estimate for low-income and middle-income countries (LMICs; 3·2 per 100 person-years, 95% CI 2·2–4·6),^12^ which aligns with modelled estimates for sub-Saharan Africa (3·4 per 100 person-years).^16^ For countries in sub-Saharan Africa with missing incidence estimates for men who have sex with men (16 of 26) and female sex workers (21 of 28), we used subregional (eastern and southern Africa or western and central Africa) estimates from existing systematic reviews.^14,15^ For men who have sex with men, subregional estimates have already been published,^14^ whereas we calculated subregional estimates for female sex workers using data from an existing systematic review.^15^ For transgender women in sub-Saharan Africa, we used country-specific estimates identified through our ongoing global review of HIV incidence among men who have sex with men and transgender women (appendix 2 pp 4–6). For countries without estimates for transgender women (12 of 15), we applied a pooled incidence rate ratio (IRR; 2·40, 95% CI 1·38–4·19, based on six studies identified in our review that estimated incidence in both men who have sex with men and transgender women) to the corresponding country-specific HIV incidence estimate for men who have sex with men. For non-key populations, we used age-stratified and sex-stratified estimates of HIV incidence from existing nationally representative studies or, when unavailable, subregional estimates from a systematic review.^17^ All country-specific inputs are in appendix 2 (pp 4–10).

We used PEPFAR monitoring and evaluation data on the number of people who returned for an oral PrEP follow-up visit during July–September, 2024, for each key population group and for men and women aged 15–24 years or 25 years and older (PEPFAR, personal communication; appendix 2 pp 4–10). We assume these numbers reflect the numbers of individuals receiving PrEP at the time of the US Government’s stop-work order. We did not use data on the number of new PrEP initiates as studies suggest many of these initiates do not return for refills and are thought not to use PrEP.^18,19^ Although PrEP is available for key populations in Uganda, data were not reported for these populations in 2024 or 2023, so we estimated the number of people in each key population on PrEP through the proportions of men and women using PrEP that were a member of each key population in 2022.

Based on available evidence, we assumed different levels of oral PrEP effectiveness for different populations (appendix 2 p 12). For men who have sex with men and transgender women, we assumed an effectiveness of 75·0% (95% CI 39·0–90·0), based on pooled estimates for men who have sex with men.^20^ For people who inject drugs, we assumed an effectiveness of 48·9% (9·6–72·2), based on the Bangkok Tenofovir study.^21^ For female sex workers, we took a similar approach to our modelling of PrEP for female sex workers in South Africa.^22^ Using studies identified in a systematic review of PrEP adherence among female sex workers published in 2024,^23^ we pooled estimates of the proportion of female sex workers with high levels of adherence (based on plasma drug-level testing) to PrEP after 3 months (49%, 95% CI 32–66). For these female sex workers, we applied an estimated efficacy of PrEP for highly adherent populations based on data from a recent systematic review (79·9%, 67·2–87·6),^22^ whereas we assumed no efficacy for female sex workers with undetectable drug levels or low adherence. This approach gave an estimate for the overall effectiveness of PrEP for female sex workers of 38·8% (95% CI 27·0–51·4). For non-key population groups, we pooled sex-stratified estimates from randomised controlled trials for heterosexual men and women from a systematic review published in 2023.^24^ This systematic review gave effectiveness estimates of 69% (56 to 78) for non-key population men and 31% (–10 to 57) for non-key population women, nalthough we assumed no detrimental effect of PrEP in our model projections.

See Online for appendix 2

We used subregional estimates of the projected HIV transmission rates for different population groups from an ongoing mathematical model comparison analysis of 15 HIV models in Africa^25^ (estimates in appendix 2 p 12). Estimates were unavailable for transgender women and people who inject drugs. For transgender women, we applied our pooled HIV IRR for transgender women compared with men who have sex with men to the transmission rates for men who have sex with men. For people who inject drugs, we estimated country-specific transmission rates that would produce the projected number of new HIV infections occurring among people who inject drugs (based on the assumed HIV incidence rate), assuming that all transmission occurred within this population.

All parameters had uncertainty distributions associated with them (appendix 2 p 3), based on the studies they were derived from. For each country and subpopulation, 100 000 parameter sets were sampled from these distributions and used in the model analyses.

### Model analyses

For each subpopulation, we first estimated the country-level proportion of HIV-negative people receiving PrEP funded through PEPFAR (referred to as the coverage). We then calculated, by country, the relative increase in new HIV infections that would occur in each subpopulation if PEPFAR-funded PrEP is suspended for a year. We also estimated the overall coverage and relative increase in new infections for each subpopulation across all PEPFAR countries.

We then used HIV incidence estimates to estimate the absolute increase in new primary HIV infections that would occur in each subpopulation during a year if PEPFAR-funded PrEP was stopped in each country. By summing across countries, we also estimated the total number of new HIV infections that would occur overall and among each subpopulation across PEPFAR countries. We then used estimates of HIV transmission rates to estimate the number of secondary infections that could occur during the next 5 years resulting from a 1-year pause in PrEP funding. The variation across the different parameter sets was used to produce a median and 95% uncertainty intervals (95% UIs; 2·5–97·5 percentile range across all model runs) for all modelled outcomes.

We did a sensitivity analysis in which we considered, among men using PrEP, the potential under-reporting of sex with other men (ie, under-reporting of being a man who has sex with men). Based on internal PEPFAR discussions, in countries where no men on PrEP reported having sex with men, we assumed that 80% of non-key population males on PrEP were men who have sex with men; whereas in countries where some men on PrEP did report having sex with men, we assumed that 50% of men who did not report having sex with men were in fact men who have sex with men. All model analyses were done with MATLAB (version R2024b).

### Role of the funding source

The funder of the study had no role in study design, data collection, data analysis, data interpretation, or writing of the report.

## Results

Overall, our data suggested that at the end of 2024, 719 384 individuals who were not breastfeeding or pregnant received PEPFAR-funded PrEP across 28 countries in sub-Saharan Africa. This number included 205 868 members of key populations: 12 971 people who inject drugs across 15 countries, 52 548 men who have sex with men across 26 countries, 3666 transgender women across 16 countries, and 136 683 female sex workers across 28 countries. The number of non-key population men and women using PrEP by age are included in appendix 2 (pp 2–3). These data mean that overall 4·4% (95% UI 3·9–4·9) of HIV-negative people in key populations were estimated to be receiving oral PrEP through PEPFAR, with coverages of 2·6% (2·0–3·4) among people who inject drugs, 2·7% (2·0–3·7) among transgender women, 4·0% (3·5–4·5) among men who have sex with men, and 5·0% (4·3–5·9) among female sex workers.

**Figure 1:**
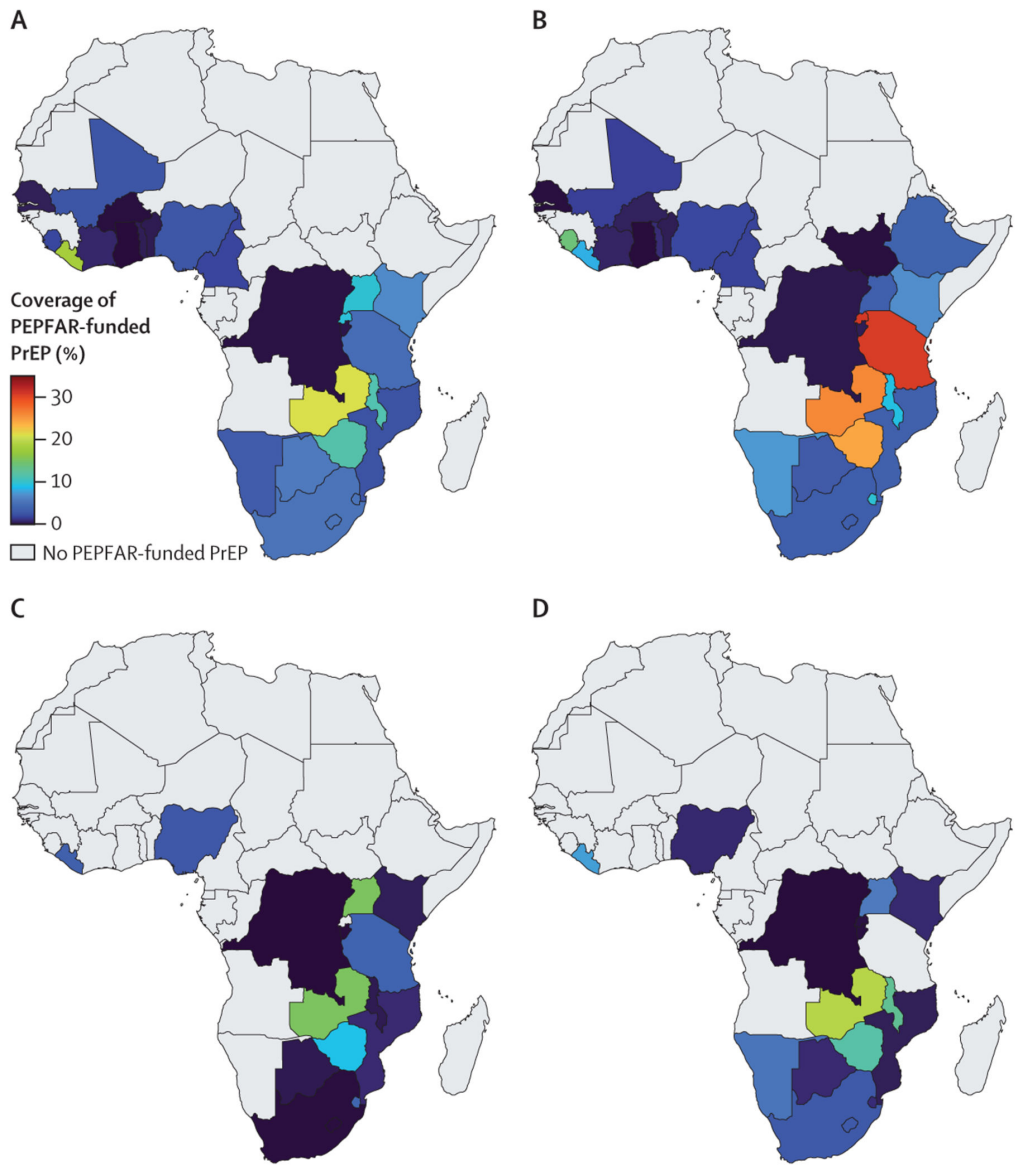
Coverage of PEPFAR-funded PrEP among HIV-negative people from each key population in sub-Saharan Africa Panels show the coverage for the key populations of men who have sex with men (A), female sex workers (B), people who inject drugs (C), and transgender women (D). Shading represents the median proportion of HIV-negative people in key populations that are receiving PEPFAR-funded PrEP. Grey shading shows countries without PEPFAR-funded PrEP. Country-level results with uncertainty intervals are presented in the appendix (pp 13–14). PEPFAR=President’s Emergency Plan for AIDS Relief. PrEP=pre-exposure prophylaxis.

**Figure 2:**
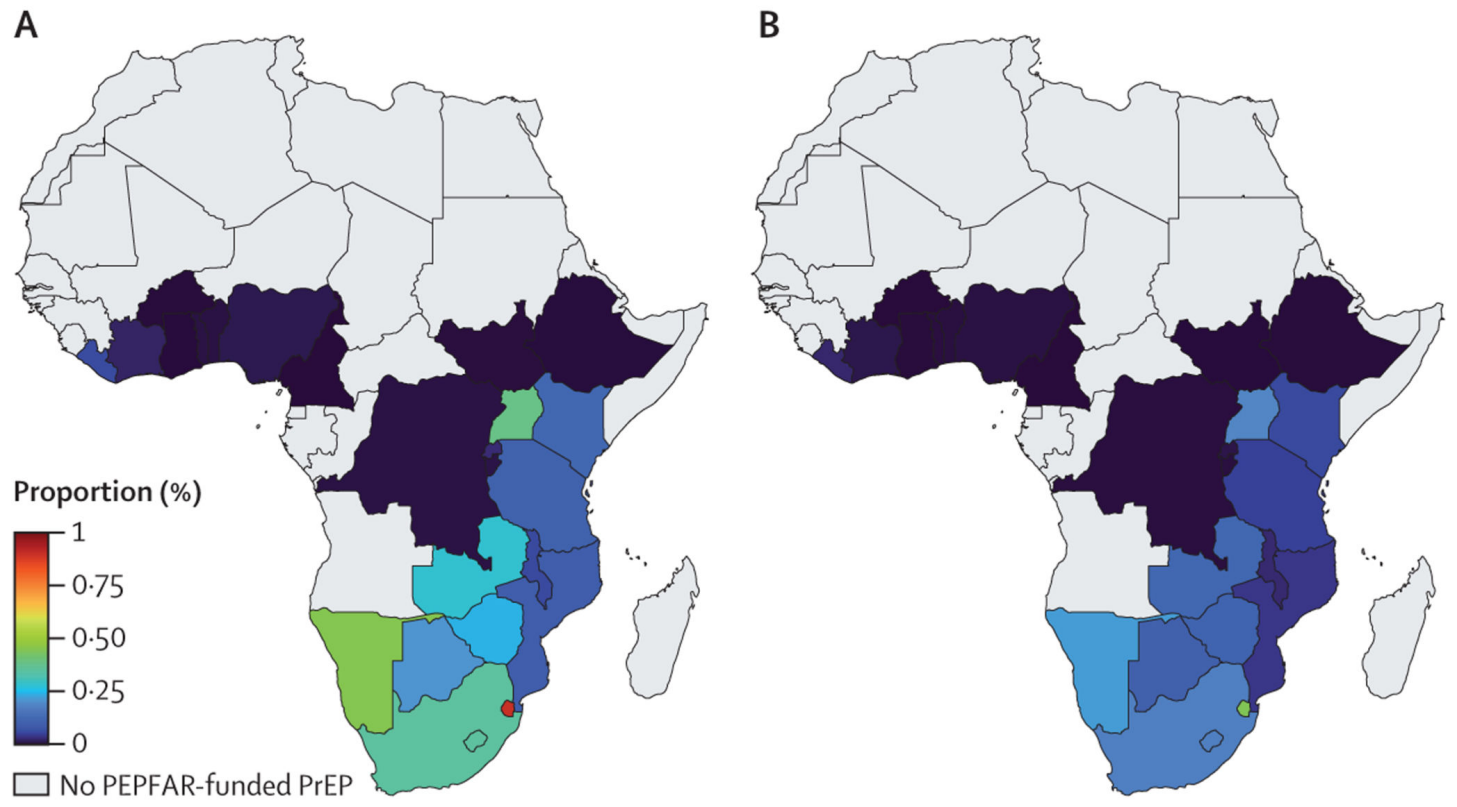
Coverage of PEPFAR-funded PrEP and impact of removing this PrEP in non-key populations in sub-Saharan Africa (A) 2024 coverage of PEPFAR-funded PrEP among HIV-negative non-key populations in sub-Saharan Africa. (B) Relative increase of new (primary) HIV infections when removing provision of PEPFAR-funded PrEP for a year for non-key populations. Shading represents median values; 95% uncertainty intervals for each country are presented in the appendix (pp 13–16). Grey shading shows countries without PEPFAR-funded PrEP. PEPFAR=President’s Emergency Plan for AIDS Relief. PrEP=pre-exposure prophylaxis.

For each key population, there was considerable heterogeneity in country-specific coverage levels (figure 1); for example, among men who have sex with men, the median coverage ranged from less than 1·0% in ten countries to 21·0% in Zambia. Conversely, 0·1% (95% UI 0·1 to 0·1) of HIV-negative non-key populations were receiving oral PrEP through PEPFAR, less than 0·1% (<0·1 to <0·1) in men and 0·1% (0·1 to 0·1) in women, with coverage greatest in women aged 15–24 years (0·2% [95% UI 0·2 to 0·2]; figure 2).

**Figure 3:**
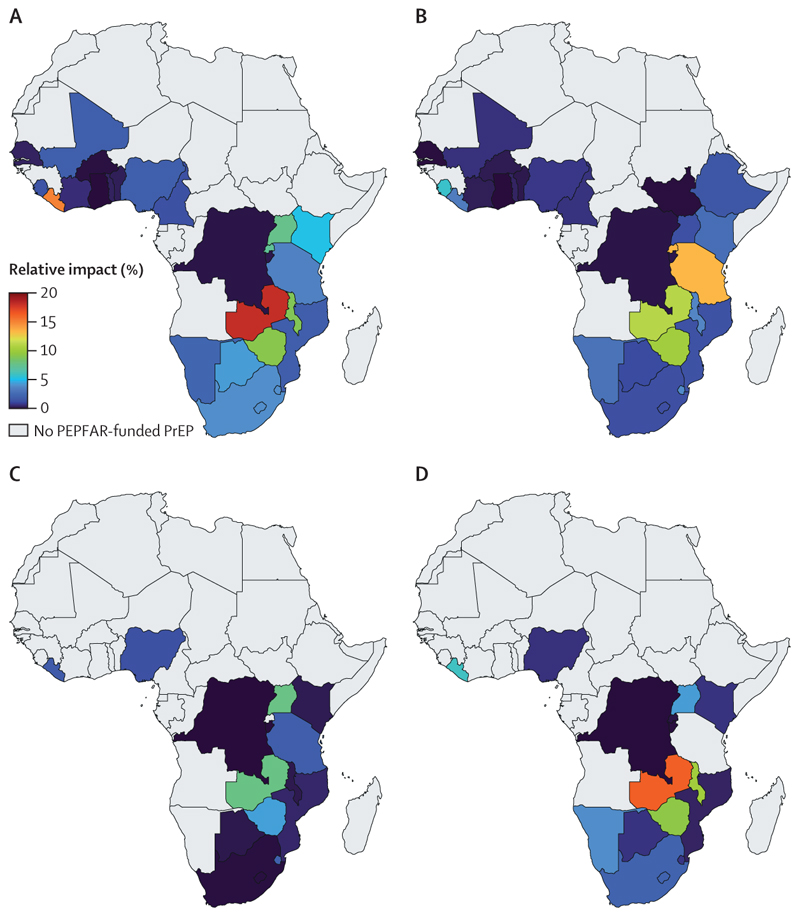
Relative impact on new (primary) HIV infections of removing provision of PEPFAR-funded PrEP for a year for each key population Panels show the impact for the key populations of men who have sex with men (A), female sex workers (B), people who inject drugs (C), and transgender women (D). Shading represents the median relative increase in new HIV infections resulting from removing PEPFAR-funded PrEP. Grey shading shows countries where PEPFAR did not provide PrEP. Country-level results with uncertainty intervals are presented in the appendix (pp 15–16). PEPFAR=President’s Emergency Plan for AIDS Relief. PrEP=pre-exposure prophylaxis.

**Figure 4:**
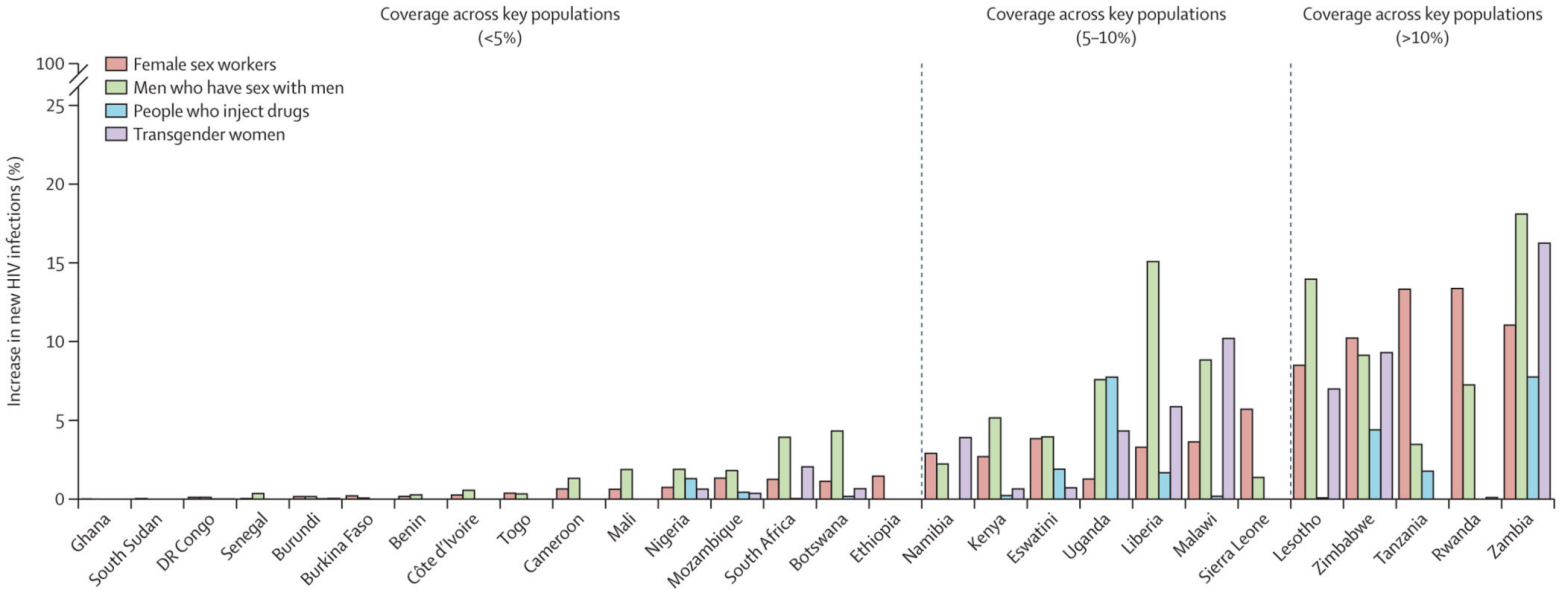
Relative impact on new (primary) HIV infections of removing provision of PEPFAR-funded PrEP for a year for each key population by country Bars represent the median relative increase in new HIV infections resulting from removing PEPFAR-funded PrEP. Country-level results with uncertainty intervals are presented in the appendix (pp 15–16). Countries are ordered based on the coverage of PrEP funded by PEPFAR across all key populations for which it is provided. PEPFAR=President’s Emergency Plan for AIDS Relief. PrEP=pre-exposure prophylaxis.

Overall, across all countries where PEPFAR funds PrEP, removing this provision of oral PrEP for a year could lead to a median 2·1% (95% UI 1·5 to 2·8) increase in new primary HIV infections across all key populations, 0·8% (0·3 to 1·5) increase in people who inject drugs, 2·2% (1·3 to 3·0) increase in men who have sex with men, 1·4% (0·8 to 2·3) increase in transgender women, 2·9% (1·8 to 4·4) increase in female sex workers, and 0·1% (<0·1 to 0·1) increase in non-key populations. The country-level impact of removing PEPFAR’s provision of PrEP depends on existing coverage and is largest in the countries with the highest estimated PrEP coverages (>15%; figures 1–4). For example, removal of PrEP in Zambia would increase new primary HIV infections by 18·1% (95% UI 9·7–33·2) in men who have sex with men, 16·3% (7·1–49·6) in transgender women, 11·1% (5·9–21·5) in female sex workers, and 7·8% (2·5–17·1) in people who inject drugs. In high coverage countries, the number of new HIV infections is projected to increase by at least 5% in eight countries for men who have sex with men, two countries for people who inject drugs, five countries for transgender women, six countries for female sex workers, but in no countries for non-key populations. Conversely, in countries with low coverage (<2%), removal of PEPFAR’s provision of PrEP would result in a less than 1% increase in new primary HIV infections in eight countries for men who have sex with men, eight countries for transgender women, 12 countries for female sex workers, eight countries for people who inject drugs, and all 28 countries for non-key populations.

**Figure 5:**
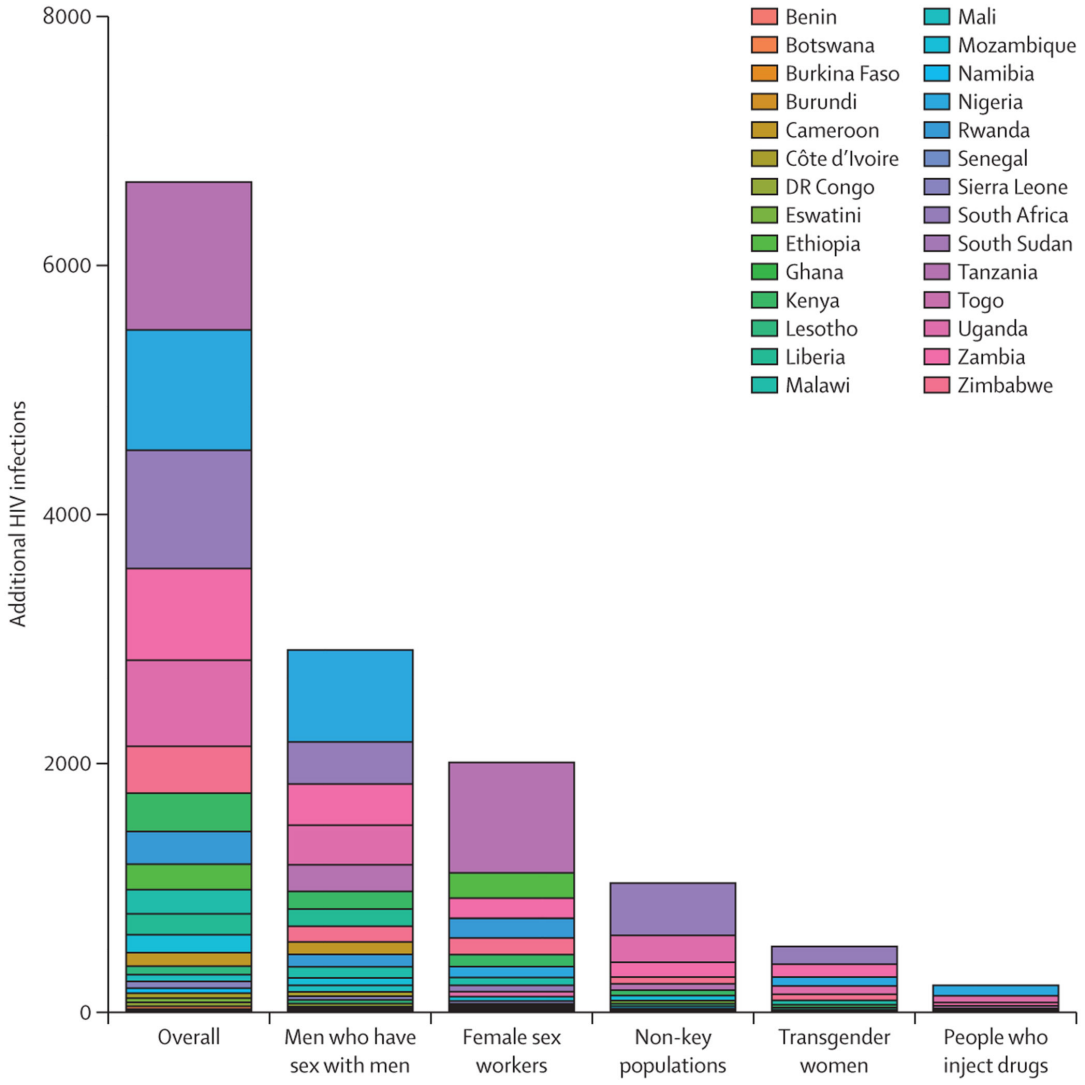
Increase in new (primary) HIV infections during a year of removing provision of PEPFAR-funded PrEP for each key population and non-key populations Bars stack the median number of additional new infections projected to occur for each country. Country-level results with uncertainty intervals are presented in the appendix (pp 17–18). PEPFAR=President’s Emergency Plan for AIDS Relief. PrEP=pre-exposure prophylaxis.

**Figure 6:**
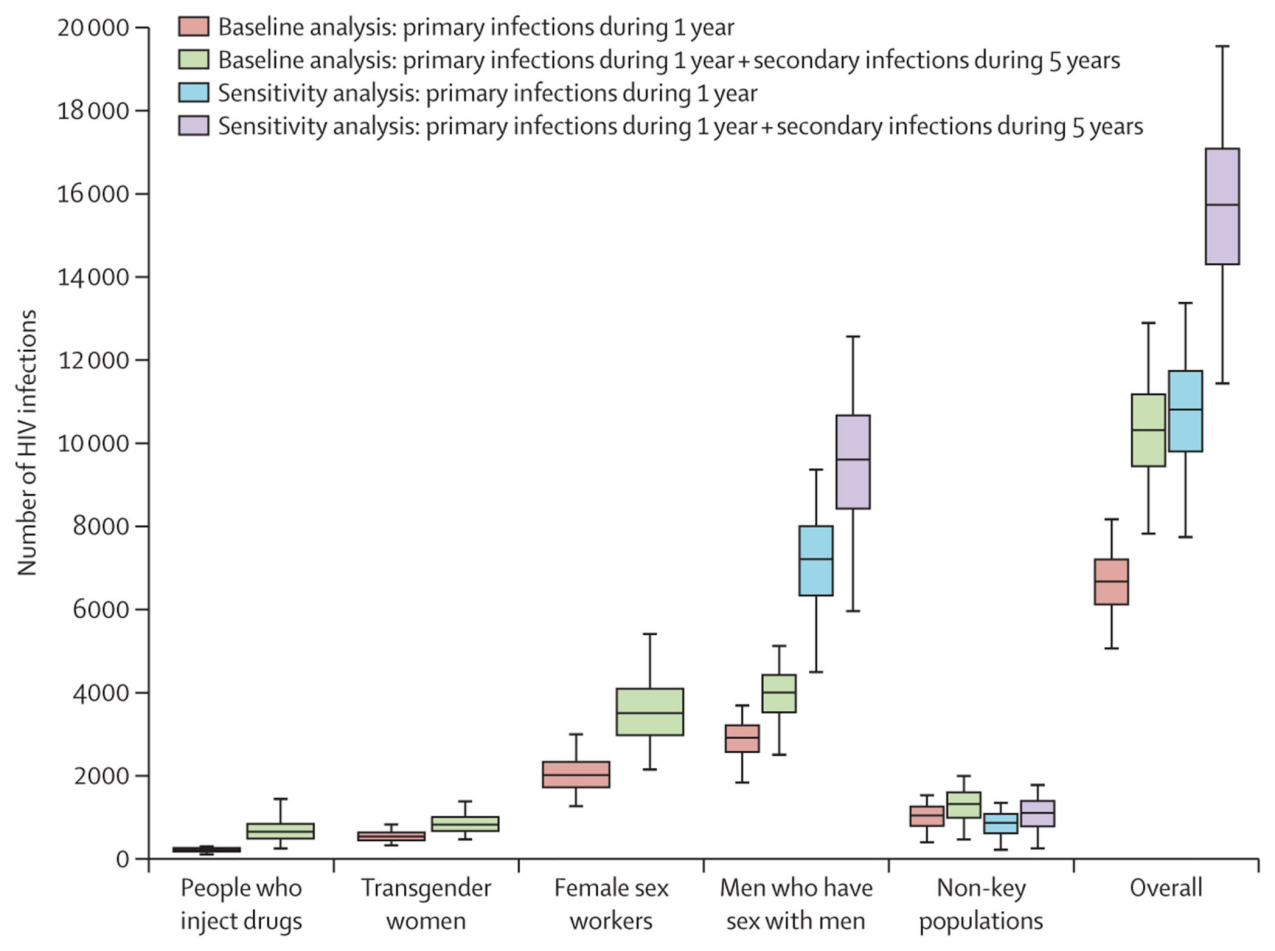
Overall increase in new HIV infections due to removing provision of PEPFAR-funded PrEP for a year The box plots show the uncertainty in the model projections (middle line is the median, limits of boxes are the 25% and 75% percentiles, and whiskers are the 2·5% and 97·5% percentiles). The baseline analysis uses the reported numbers of men who have sex with men on PrEP; the sensitivity analysis assumes 50–80% of non-key population men on PrEP have sex with men. PEPFAR=President’s Emergency Plan for AIDS Relief. PrEP=pre-exposure prophylaxis.

Overall, we estimate that the removal of PEPFAR’s provision of PrEP for a year would lead to 6671 (95% UI 5032–8192) additional new primary HIV infections (figures 5, 6), distributed as follows: 214 (95% UI 73–314) additional infections in people who inject drugs, 2909 (1802–3709) in men who have sex with men, 526 (293–844) in transgender women, and 2006 (1232–3014) in female sex workers, totalling 5663 (4146–7074) in key populations and 1035 (364–1547) in non-key populations. We estimate that two-thirds (68%) of the additional primary HIV infections would occur in five countries: Tanzania (1165 infections, 95% UI 693–1885), Nigeria (946 infections, 647–1192), South Africa (931 infections, 554–1284), Zambia (721 infections, 469–1111), and Uganda (679 infections, 432–1030). In addition, we estimate that the secondary impact of these additional new primary infections could give rise to a further 3617 (95% UI 2585–5013) secondary infections during the next 5 years, resulting in an estimated total of 10 313 (7796–12 921) new HIV infections (ie, primary and secondary infections).

In sensitivity analyses, in which we accounted for potential under-reporting of men who have sex with men among men on PrEP, we estimate that removing PrEP for a year could lead to 10 811 (95% UI 7714–13 401) additional primary HIV infections during that year (62·1% higher than our baseline projections) or 15 739 (95% UI 11 414–19 585) additional primary and secondary HIV infections during the next 5 years (52·6% higher than our baseline projections). Country-level results are given in the appendix (pp 13–24).

## Discussion

Our analysis shows the important negative impact that could result from ceasing PEPFAR’s funding of PrEP for a year. In 2024, PEPFAR was providing oral PrEP to 719 384 individuals who are not pregnant or breastfeeding, including 205 868 key population individuals in sub-Saharan Africa, with provision being highest in female sex workers (136 683 people) and women aged 15–24 years (153 838 people). If this provision was removed for a year, then we estimate that 6671 additional new primary infections could occur among individuals that would have been using PrEP, the majority of which (5663) would occur among key populations. This number equates to a 2·1% increase in infections among key populations in sub-Saharan Africa. The impact is highest in men who have sex with men, with 2909 (2·2% increase) additional new infections, and female sex workers, with 2006 (2·9% increase) additional new infections. When also accounting for secondary HIV transmissions resulting from these newly infected individuals and possible under-reporting of men who have sex with other men among men on PrEP, we estimate that a 1-year pausing of PEPFAR-funded PrEP could result in 15 739 new HIV infections during the next 5 years.

These projections add to an emerging body of studies evaluating the negative impacts of cuts in HIV funding. These studies have shown that potential cuts in antiretroviral therapy (ART) for 90 days could result in 100 000 excess deaths in sub-Saharan Africa,^26^ increasing to 7·5–13·4 million additional AIDS-related deaths by 2030 if disruptions in ART are sustained.^27^ Conversely, the complete termination of PEPFAR support could result in 565 000 additional infections over the course of 10 years in South Africa^28^ or, if combined with further reductions in international aid from the USA, the UK, France, Germany, and the Netherlands, could lead to 4·43–10·75 million additional infections across all LMICs during the next 5 years.^29^ Additionally, an online tracker is using previous model results to produce simple projections of the health and HIV transmission consequences of a halt in the PEPFAR provision of HIV treatment.^30^ Our study adds to this emerging literature by being the first, to our knowledge, analysis to estimate the detrimental impact of ceasing PEPFAR’s provision of PrEP among key populations and other population groups, giving predictions for each key population in each country in sub-Saharan Africa. Key populations are especially vulnerable to foreign aid cuts, which have been further exacerbated by the Trump administration’s executive orders targeting gender minorities and its disregard for evidence-based policies supporting key populations.^31^

The strength of our analysis is in producing novel impact projections for all key populations and other subpopulations in all countries in sub-Saharan Africa where PEPFAR funds PrEP. We use the best available data from systematic reviews published in the past couple of years to parametrise sizes of key populations, HIV prevalence and HIV incidence for each subpopulation, and data on the actual number of people using oral PrEP from PEPFAR in 2024.

Limitations of this study include making short-term projections of the additional new HIV infections arising from removing PEPFAR funding for PrEP. Although we used estimates of the HIV transmission rates among different groups to estimate the number of secondary infections that could arise during the next 5 years, our results do not fully account for the elevated infectiousness of people with recent infections after the removal of PrEP or the full chain of transmissions that could arise over time. As such, our results might be conservative, with the impact of removing PEPFAR-funded PrEP likely to increase over longer time periods. It is also important to note that our modelling estimates less than one secondary infection resulting from each primary infection during the next 5 years. This low amount of secondary transmission is to be expected in the context of contracting HIV epidemics in Africa, which suggests that the overall number of onward transmissions per person living with HIV is less than one during their lifetime.

We also assumed that the number of people receiving PrEP funded by PEPFAR would not have increased in 2025 without the funding cuts. Data suggest that, globally, the number of individuals retained on PrEP funded by PEPFAR has increased over time, including by 41% from 2023 to 2024, from 578 024 people to 815 107. If we assumed that the numbers on PrEP would have increased at the same rate in 2025, then removing PEPFAR-funded PrEP in 2025 would result in 9407 additional primary HIV infections in sub-Saharan Africa and 14 543 in total when secondary infections are also included.

The PrEP use data we used are based on individuals self-identifying as specific key populations. This self-identification is likely to underestimate the real number of each key population using PrEP because individuals might not want to identify as such groups due to stigma or social desirability bias. This likely under-reporting makes our results conservative. Indeed, in our sensitivity analysis in which we assumed that men on PrEP under-report sex with other men, our 1-year projection increased by 62% and the 5-year projection by 53%. There is also uncertainty in our parameter estimates, especially in the population size estimates for each key population. It is likely that some individuals might not want to identify with specific key populations because their behaviours are criminalised or stigmatised. This issue is highlighted in some countries where the population size estimates of specific key populations are small. In these settings, our estimates of the relative increase in HIV infections resulting from removing PrEP among these groups might be overestimated, although it should not affect our estimates of the absolute number of infections.

Uncertainty also exists in our HIV incidence estimates among key populations, with many countries not having data, and for those countries with data, the studies are usually subnational or not necessarily recent (ie, older than 5 years). For men who have sex with men, these uncertainties were accounted for in a recent systematic review^14^ that used Bayesian modelling to estimate the HIV incidence at the country and regional level. However, for people who inject drugs, transgender women, and female sex workers, the data are sparser. When missing, we used pooled subregional (for eastern and southern Africa, and western and central Africa) estimates for female sex workers, a pooled estimate for people who inject drugs across LMICs, and adjusted men who have sex with men estimates to generate estimates for transgender women using a pooled IRR from sub-Saharan Africa. Consequently, our absolute infection estimates will be uncertain for these groups except for the countries with data. For non-key populations, we used HIV incidence estimates from nationally representative surveys conducted since 2016. However, 11 countries did not have data, so we used pooled subregional estimates. The pooled estimates did not increase the uncertainty in our projections because they accounted for less than 2% of non-key populations on PrEP.

Our HIV prevalence estimates for each subpopulation are less uncertain as this metric is typically estimated for most key populations and other groups through national level biobehavioural surveys. However, there is still uncertainty in HIV prevalence estimates for people who inject drugs and transgender women because fewer surveys have been done in these groups in sub-Saharan Africa. Lastly, for both the estimation of the relative and absolute impact of removing PEPFAR-funded PrEP for each subpopulation, the model assumes that HIV-negative individuals receiving PrEP have similar risk behaviour to the remainder of the HIV-negative population not on PrEP. It is possible that PrEP is offered to individuals with higher behavioural risk than those not on PrEP or, alternatively, that more vulnerable individuals might not attend services in which PrEP is offered, meaning that individuals offered PrEP could be at lower risk. It is difficult to determine the magnitude and direction of this bias, as existing evidence on this is conflicted,^23^ and so we did not account for it in our modelling. Future biobehavioural surveys could further assess this bias by comparing the risk behaviour of people offered PrEP or on PrEP with those not offered or on PrEP, and future modelling should incorporate heterogeneity in both HIV risk and PrEP uptake and outcomes.

Ceasing PEPFAR’s funding for PrEP in sub-Saharan Africa will stop approximately 700 000 individuals from using oral PrEP, including 200 000 key population individuals. This reduction in people who can access PrEP could lead to an estimated 6671 additional primary HIV infections in the next year, with most of these infections (5663) occurring among key populations. Although this is an important impact, it equates to less than a 3% increase in HIV infections among key populations, primarily due to the low coverage of PrEP among these groups in most countries in sub-Saharan Africa. It is therefore crucial that these PrEP services are continued and expanded as PEPFAR is the primary source of support for PrEP in sub-Saharan Africa. Such an expansion not only requires reversing funding cuts but also increasing previous funding and other funding through domestic resources and from alternative donors to sustain and increase access. Unfortunately, in many parts of the world, it is unlikely that national governments will substantially increase support for key population programming, given their historical neglect of these groups and the unfavorable policy environments that often restrict or undermine such efforts.^32^ Although some countries have started to use domestic funds to maintain HIV services,^7^ it is uncertain whether this includes the provision of PrEP. If funding for key populations does not resume, then the impact is likely to be greater than we estimate because funding for other prevention services among key populations has also halted^7^ or been severely affected.^33^ This reduction in HIV prevention services is likely to negate the progress that has been achieved in tackling HIV transmission among key populations, which has been crucial for controlling the HIV epidemics in sub-Saharan Africa.^34^

## Supplementary Material

appendix 1

appendix 2

## Data Availability

The model code and projections for this study will be shared with interested parties based on a suitable analysis plan, which will be reviewed by the authors.

## Research in context

### Evidence before this study

In January, 2025, the US Government issued a directive, pausing all foreign aid programmes. This included a 90-day pausing of the US President’s Emergency Plan for AIDS Relief (PEPFAR) funding for HIV pre-exposure prophylaxis (PrEP), except for pregnant and breastfeeding women. PEPFAR-funded PrEP accounts for more than 90% of global initiations on to oral HIV PrEP. We searched PubMed for articles published from Jan 1, 2025, to June 19, 2025, with the following terms: (model* AND HIV AND 2025 AND (PEFPAR OR fund*)). We also asked coauthors for suitable articles. There was no language restriction. We identified five modelling analyses that project the impact on HIV of various aspects of the US funding cuts for low-income and middle-income countries. A 90-day disruption to antiretroviral therapy for HIV is estimated to result in 100 000 excess deaths in sub-Saharan Africa, with another modelling analysis projecting that a longer-term disruption could lead to 7·5–13·4 million AIDS-related deaths by 2030. At the country level, a complete termination of PEPFAR support in South Africa is estimated to result in 565 000 additional HIV infections during the next 10 years, and a partial ceasing of PEPFAR-funded services in Rwanda could result in 20 000–92 000 additional adult HIV infections and 10 000–51 200 additional deaths in 10 years. Lastly, a discontinuation of PEPFAR funding alongside an additional 20% reduction in other international aid is estimated to result in 4·4–10·8 million additional infections in the next 5 years across all low-income and middle-income countries through reductions in HIV treatment and prevention interventions. Although highly relevant, none of these analyses have evaluated the impact of funding cuts in key populations, who are especially marginalised and at heightened risk of acquiring HIV, and none evaluated the specific impact of ceasing PEPFAR’s provision of oral PrEP. Although waivers have been given for HIV treatment and other interventions, it is unlikely that a return in PEPFAR funding for HIV prevention interventions, and specifically PrEP, will occur, especially among key populations.

### Added value of this study

We estimated the impact of a 1-year funding pause for oral PrEP in key populations and non-key populations in 28 countries in sub-Saharan Africa where PEPFAR provided PrEP. Removing PEPFAR’s provision of PrEP for a year could lead to 6671 additional primary infections, with 5663 of these occurring among key populations—a 2·1% increase from current rates. This impact will be greatest among men who have sex with men, with 2909 additional new primary infections, and female sex workers, with 2006 additional new primary infections. Most (68%) of the impact from removing PrEP (in terms of primary infections) will occur in Tanzania (1165 infections), Nigeria (946), South Africa (931), Zambia (721), and Uganda (679). When accounting for secondary infections, a 1-year removal of PrEP could lead to 10 313 additional infections during the next 5 years in the overall population.

### Implications of all the available evidence

Our study adds to the growing evidence projecting the large, detrimental impact that cuts to PEPFAR funding could have on HIV outcomes in sub-Saharan Africa. If a return in PEPFAR funding without the restrictions imposed since January, 2025 does not occur, then urgent measures are needed to mitigate against this impact. These measures should include identifying funding through alternative international donors or domestic government budgets. Additionally, given the low coverages of oral PrEP identified in our study, additional funding is also needed to expand PrEP to increase its population-level impact.

## References

[1] US President’s Emergency Plan for AIDS Relief (2024). Latest global program results.

[2] The Kaiser Family Foundation (2025). The US President’s Emergency Plan for AIDS Relief (PEPFAR).

[3] UNAIDS Progress at the halfway mark to the 2025 milestones.

[4] Korenromp EL, Sabin K, Stover J (2024). New HIV infections among key populations and their partners in 2010 and 2022, by world region: a multisources estimation. J Acquir Immune Defic Syndr.

[5] Ratevosian J, Millett G, Honermann B (2025). PEPFAR under review: what’s at stake for PEPFAR’s future. Lancet.

[6] Lankiewicz E, Sharp A, Drake P (2025). Early impacts of the PEPFAR stop-work order: a rapid assessment. J Int AIDS Soc.

[7] UNAIDS (2025). Impact of US funding cuts on the global AIDS response. Weekly update—10 March 2025.

[8] US Department of State of Global Health Security and Diplomacy (2025). HIV Care & Treatment and Prevention of Mother to Child Transmission Activities Approved Under PEPFAR Limited Waiver.

[9] Jerving S (2025). Senate blocks $400M cut to, PEPFAR., but it’s a shell of its former self.

[10] Degenhardt L, Webb P, Colledge-Frisby S (2023). Epidemiology of injecting drug use, prevalence of injecting-related harm, and exposure to behavioural and environmental risks among people who inject drugs: a systematic review. Lancet Glob Health.

[11] Stevens O, Sabin K, Anderson RL (2024). Population size, HIV prevalence, and antiretroviral therapy coverage among key populations in sub-Saharan Africa: collation and synthesis of survey data, 2010–23. Lancet Glob Health.

[12] Artenie A, Stone J, Fraser H (2023). Incidence of HIV and hepatitis C virus among people who inject drugs, and associations with age and sex or gender: a global systematic review and meta-analysis. Lancet Gastroenterol Hepatol.

[13] Artenie A, Perry R, Mahaso M (2024). HIV incidence and factors associated with HIV risk among people who inject drugs engaged with harm-reduction programmes in four provinces in South Africa: a retrospective cohort study. Lancet HIV.

[14] Stannah J, Soni N, Lam JKS (2023). Trends in HIV testing, the treatment cascade, and HIV incidence among men who have sex with men in Africa: a systematic review and meta-analysis. Lancet HIV.

[15] Jones HS, Anderson RL, Cust H (2024). HIV incidence among women engaging in sex work in sub-Saharan Africa: a systematic review and meta-analysis. Lancet Glob Health.

[16] Stevens O, Anderson R, Stover J (2024). Comparison of empirically derived and model-based estimates of key population HIV incidence and the distribution of new infections by population group in sub-Saharan Africa. J Acquir Immune Defic Syndr.

[17] Rosenberg NE, Shook-Sa BE, Liu M (2023). Adult HIV-1 incidence across 15 high-burden countries in sub-Saharan Africa from 2015 to 2019: a pooled analysis of nationally representative data. Lancet HIV.

[18] Kagaayi J, Batte J, Nakawooya H (2020). Uptake and retention on HIV pre-exposure prophylaxis among key and priority populations in south-central Uganda. J Int AIDS Soc.

[19] Mugwanya KK, Palayew A, Schaafsma T (2023). Patterns of PrEP continuation and coverage in the first year of use: a latent class analysis of a programmatic PrEP trial in Kenya. J Int AIDS Soc.

[20] Murchu E, Marshall L, Teljeur C (2022). Oral pre-exposure prophylaxis (PrEP) to prevent HIV: a systematic review and meta-analysis of clinical effectiveness, safety, adherence and risk compensation in all populations. BMJ Open.

[21] Choopanya K, Martin M, Suntharasamai P (2013). Antiretroviral prophylaxis for HIV infection in injecting drug users in Bangkok, Thailand (the Bangkok Tenofovir Study): a randomised, double-blind, placebo-controlled phase 3 trial. Lancet.

[22] Stone J, Bothma R, Gomez GB (2023). Impact and cost-effectiveness of the national scale-up of HIV pre-exposure prophylaxis among female sex workers in South Africa: a modelling analysis. J Int AIDS Soc.

[23] Mpirirwe R, Segawa I, Ojiambo KO (2024). HIV pre-exposure prophylaxis uptake, retention and adherence among female sex workers in sub-Saharan Africa: a systematic review. BMJ Open.

[24] Chou R, Spencer H, Bougatsos C, Blazina I, Ahmed A, Selph S (2023). Preexposure prophylaxis for the prevention of HIV: updated evidence report and systematic review for the US Preventive Services Task Force. JAMA.

[25] Silhol R, Booton RD, Mitchell KM (2025). Identifying priority populations for HIV interventions using acquisition and transmission indicators: a combined analysis of 15 mathematical models from 10 African countries. medRxiv.

[26] Tram KH, Ratevosian J, Beyrer C (2025). By executive order: the likely deadly consequences associated with a 90-day pause in PEPFAR funding. J Int AIDS Soc.

[27] Cluver L, Makangila G, Hillis S (2025). Protecting Africa’s children from extreme risk: a runway of sustainability for PEPFAR programmes. Lancet.

[28] Gandhi AR, Bekker LG, Paltiel AD (2025). Potential clinical and economic impacts of cutbacks in the President’s Emergency Plan for AIDS Relief Program in South Africa: a modeling analysis. Ann Intern Med.

[29] Brink DT, Martin-Hughes R, Bowring AL (2025). Impact of an international HIV funding crisis on HIV infections and mortality in low-income and middle-income countries: a modelling study. Lancet HIV.

[30] Nichols BE, Geng EH, Moakley E (2025). Rapid development of an online tracker to communicate the human impact of abruptly halting PEPFAR support. J Int AIDS Soc.

[31] Rose M (2025). Resisting the freeze on PEPFAR: a fight to protect LGBTQ+ lives and global health progress.

[32] Nkengasong J, Ratevosian J (2023). Legal and policy barriers for an effective HIV/AIDS response. Lancet.

[33] International Network of People Who Use Drugs (2025). The human cost of policy shift: the fallout of US foreign aid cuts on harm reduction programming and people who use drugs.

[34] Stone J, Mukandavire C, Boily MC (2021). Estimating the contribution of key populations towards HIV transmission in South Africa. J Int AIDS Soc.

