# Supplementary material for: The impact of cuts in the US President’s Emergency Plan for AIDS Relief funding for HIV pre-exposure prophylaxis in sub-Saharan Africa: a modelling study
Source: Lancet HIV. Author manuscript; Available in PMC 2026 Mar 18. (PMC7618903; doi:10.1016/S2352-3018(25)00192-4)
Supplement: appendix 1 [file EMS212306-supplement-appendix_1.pdf]

THE LANCET
HIV

Supplementary appendix 1

This translation in French was submitted by the authors and we reproduce it as supplied. It has not been peer reviewed. *The Lancet*'s editorial processes have only been applied to the original in English, which should serve as reference for this manuscript.

Cette traduction en français a été proposée par les auteurs et nous l'avons reproduite telle quelle. Elle n'a pas été examinée par des pairs. Les processus éditoriaux du *Lancet* n'ont été appliqués qu'à l'original en anglais et c'est cette version qui doit servir de référence pour ce manuscrit.

Supplement to: Stone J, Kipkoech Mutai K, Artenie A, et al. The impact of cuts in the US President's Emergency Plan for AIDS Relief funding for HIV pre-exposure prophylaxis in sub-Saharan Africa: a modelling study. *Lancet HIV* 2025; published online Sept 10. https://doi.org/10.1016/S2352-3018(25)00192-4.

Résumé

Contexte En janvier 2025, le gouvernement américain a émis une directive suspendant tous ses programmes d'aide au développement international. Cette directive prévoyait une suspension de 90 jours de tous les financements du Plan présidentiel d'aide d'urgence à la lutte contre le sida (PEPFAR) destinés à la prophylaxie orale pré-exposition (PrEP) contre le VIH, sauf pour les femmes enceintes et allaitantes. Un retour de ce financement la PrEP apparaissait de plus en plus incertain. Notre objectif était d'estimer l'effet d'une suspension du financement de la PrEP sur les nouvelles infections à VIH en Afrique subsaharienne.

Méthodes Pour cette étude de modélisation mathématique, nous avons développé un modèle statique de transmission du VIH intégrant la PrEP, paramétré avec des estimations de la taille de la population, de la prévalence et de l'incidence du VIH, ainsi que de l'efficacité de la PrEP pour différentes sous-populations (y compris les populations clés) dans chaque pays d'Afrique subsaharienne recevant des fonds du PEPFAR pour la PrEP. Les populations clés étaient les hommes ayant des rapports sexuels avec d'autres hommes, les travailleuses du sexe, les femmes transgenres et les consommateurs de drogues injectables. Nous avons utilisé les données du PEPFAR sur le nombre de personnes issues de différentes sous-populations revenant pour recevoir la PrEP orale dans chaque pays entre juillet et septembre 2024 comme estimation du nombre de personnes utilisant la PrEP orale fournie par le PEPFAR. Pour chaque pays et sous-population, nous avons modélisé l'augmentation relative et absolue des nouvelles infections directes par le VIH résultant de la suppression de cette PrEP financée pendant un an, ainsi que le nombre d'infections secondaires pouvant résulter de ces infections directes au cours des cinq années suivantes.

Résultats Fin 2024, 719 384 personnes n'allaitant pas ou n'étant pas enceintes, dont 205 868 personnes étant membres de populations clés, ont reçu de la PrEP orale financée par le PEPFAR dans 28 pays d'Afrique subsaharienne. La proportion estimée de personnes séronégatives issues de populations clés recevant de la PrEP financée par le PEPFAR (i.e., la couverture de la PrEP) variait de 2·6 % (intervalle d'incertitude à 95 % : 2·0–3·4) chez les consommateurs de drogues injectables à 5·0 % (4·5–5·9) chez les travailleuses du sexe. La couverture estimée chez les hommes non-membres de populations clés était inférieure à 0·1 % (< 0·1 à < 0·1) et chez les femmes, elle était de 0·1 % (0·1 à 0·1). L'arrêt de la fourniture de PrEP par le PEPFAR pendant un an pourrait entraîner 6 671 (5 032–8 192) nouvelles infections directes par le VIH supplémentaires, dont 5 663 (4 146–7 074) chez les populations clés. Au sein des populations clés, cette augmentation correspond à des augmentations relatives des nouvelles infections par le VIH de 0·8 % (0·3–1·5) chez les personnes qui s'injectent des drogues, de 1·4 % (0·8–2·3) chez les femmes transgenres, de 2·2 % (1·3–3·0) chez les hommes ayant des rapports sexuels avec des hommes et de 2·9 % (1·8–4·4) chez les travailleuses du sexe. En Zambie, pays affichant la couverture de PrEP PEPFAR la plus élevée parmi toutes les populations clés, cette augmentation varie de 7·8 % (2·5–17·1) chez les consommateurs de drogues injectables à 18·1 % (9·7–33·2) chez les hommes ayant des rapports sexuels avec des hommes. En tenant compte des transmissions secondaires, nous avons estimé qu'une pause d'un an pourrait entraîner 10 313 (7 796–12 921) infections supplémentaires au cours des cinq prochaines années.

Interprétation Nos projections montrent les conséquences néfastes considérables que pourraient avoir les réductions du financement du PEPFAR. Des mesures de mitigation, notamment un financement par d'autres donateurs internationaux ou par les budgets nationaux, sont nécessaires pour maintenir et accroître les niveaux de couverture actuels de la PrEP et ainsi prévenir les conséquences néfastes des réductions du financement du PEPFAR, en particulier dans les pays à couverture élevée.
