# Supplementary material for: The impact of cuts in the US President’s Emergency Plan for AIDS Relief funding for HIV pre-exposure prophylaxis in sub-Saharan Africa: a modelling study
Source: Lancet HIV. Author manuscript; Available in PMC 2026 Mar 18. (PMC7618903; doi:10.1016/S2352-3018(25)00192-4)
Supplement: appendix 2 [file EMS212306-supplement-appendix_2.pdf]

THE LANCET
HIV

Supplementary appendix 2

This appendix formed part of the original submission and has been peer reviewed. We post it as supplied by the authors.

Supplement to: Stone J, Kipkoech Mutai K, Artenie A, et al. The impact of cuts in the US President's Emergency Plan for AIDS Relief funding for HIV pre-exposure prophylaxis in sub-Saharan Africa: a modelling study. *Lancet HIV* 2025; published online Sept 10. https://doi.org/10.1016/S2352-3018(25)00192-4.

Table of Contents

Estimating the number of non-key population men and women using PrEP 2

Parameter sampling 3

Supplementary Table 1: Model inputs for key population groups 4

Supplementary Table 2: Model inputs for non-key population groups. 7

Supplementary Table 3: Model inputs - proportion of PWID that are female 11

Supplementary Table 4: Model inputs – effectiveness of PrEP 12

Supplementary Table 5: Model inputs – HIV transmission rates for different subgroups and sub-regions of sub-Saharan Africa. 12

Supplementary Table 6: Baseline results - PrEP coverage among HIV-negatives 13

Supplementary Table 7: Baseline results - Relative impact of removing PEPFAR funded PrEP for 1-year (Primary Infections) 15

Supplementary Table 8: Baseline results - Absolute impact of removing PEPFAR funded PrEP for 1-year (Primary Infections) 17

Supplementary Table 9: Baseline results - Absolute impact of removing PEPFAR funded PrEP for 1-year (Primary Infections over 1 year and Secondary Infections over 5 years) 19

Supplementary Table 10: Sensitivity Analysis – Absolute impact of removing PEPFAR funded PrEP for 1-year (Primary Infections) 21

Supplementary Table 11: Sensitivity Analysis – Absolute impact of removing PEPFAR funded PrEP for 1-year (Primary Infections over 1 year and Secondary Infections over 5 years). 23

References 25

Estimating the number of non-key population men and women using PrEP

PEPFAR monitoring and evaluation data provides the numbers who returned for a PrEP follow-up visit during July-September 2024(1) for each key population group, pregnant and breastfeeding women, and all men and women aged 15-24 or 25+ or, in some instances, with unknown age. For each country, we use these data to estimate the number of non-key population men and women aged 15-24, 25-34 and 35-49 who are using PrEP using the below assumptions:

1. Men and women using PrEP of unknown ages are assigned to the respective 15-24 and 25+ age groups proportionately based on the age distributions of men and women using PrEP with known ages in that country.
2. Men and women using PrEP aged 25+ are assigned into the 25-34 and 35-49 age groups proportionately based on the population sizes of these age groups for each sex.
3. The proportion of PWID receiving PrEP that are female is the same as the proportion of PWID that are female in a country (Supplementary Table 3, Appendix pp 13).
4. The age distribution of male and female key populations (and pregnant and breast-feeding women) receiving PrEP is the same as the age distribution of all men and women using PrEP.

We estimate the number of non-key population men aged 15-24 ($P_{15-24}^{Non-KPM}$), 25-34 ($P_{25-34}^{Non-KPM}$) and 35-49 ($P_{35-49}^{Non-KPM}$) as:

$$P_{15-24}^{Non-KPM} = P_{15-24}^{M} + \frac{P_{15-24}^{M}}{P_{15-24}^{M} + P_{25+}^{M}}(P_U^M - P^{PWID} * (1-\tau_f) - P^{MSM} - P^{TGW})$$

$$P_{25-34}^{Non-KPM} = \frac{N_{25-34}^{M}}{N_{25-34}^{M} + N_{35-49}^{M}} * [P_{25+}^{M} + \frac{P_{25+}^{M}}{P_{15-24}^{M} + P_{25+}^{M}}(P_U^M - P^{PWID} * (1-\tau_f) - P^{MSM} - P^{TGW})]$$

$$P_{35-49}^{Non-KPM} = \frac{N_{35-49}^{M}}{N_{25-34}^{M} + N_{35-49}^{M}} * [P_{25+}^{M} + \frac{P_{25+}^{M}}{P_{15-24}^{M} + P_{25+}^{M}}(P_U^M - P^{PWID} * (1-\tau_f) - P^{MSM} - P^{TGW})]$$

Where
P_{15-24}^{M}, P_{25+}^{M} and P_U^M are the numbers of male PrEP users aged 15-24, aged 25+, or with missing age, respectively.

P^{PWID}, P^{MSM} and P^{TGW} are the numbers of PWID, MSM and TGW using PrEP, respectively.

τ_f is the proportion of PWID that are female
N_{25-34}^{M} and N_{35-49}^{M} are the population sizes of men aged 25-34 and 35-49, respectively.

Similarly, we estimate the number of non-key population women aged 15-24 ($P_{15-24}^{Non-KPF}$), 25-34 ($P_{25-34}^{Non-KPF}$) and 35-49 ($P_{35-49}^{Non-KPF}$) as:

$$P_{15-24}^{Non-KPF} = P_{15-24}^{F} + \frac{P_{15-24}^{F}}{P_{15-24}^{F} + P_{25+}^{F}}(P_U^F - P^{PWID} * \tau_f - P^{FSW} - P^{PBF})$$

$$P_{25-34}^{Non-KPF} = \frac{N_{25-34}^{F}}{N_{25-34}^{F} + N_{35-49}^{F}} * [P_{25+}^{F} + \frac{P_{25+}^{F}}{P_{15-24}^{F} + P_{25+}^{F}}(P_{U}^{F} - P^{PWID} * \tau_f - P^{FSW} - P^{PBF})]$$

$$P_{35-49}^{Non-KPF} = \frac{N_{35-49}^{F}}{N_{25-34}^{F} + N_{35-49}^{F}} * [P_{25+}^{F} + \frac{P_{25+}^{F}}{P_{15-24}^{F} + P_{25+}^{F}}(P_{U}^{F} - P^{PWID} * \tau_f - P^{FSW} - P^{PBF})]$$

Where
P_{15-24}^{F}, P_{25+}^{F} and P_{U}^{F} are the numbers of female PrEP users aged 15-24, aged 25+, or with missing age, respectively.

P^{PWID}, P^{FSW} and P^{PBF} are the numbers of PWID, FSW and pregnant and breastfeeding women using PrEP, respectively.

τ_f is the proportion of PWID that are female

N_{25-34}^{F} and N_{35-49}^{F} are the population sizes of women aged 25-34 and 35-49, respectively.

Parameter sampling

We used Lognormal distributions to sample PrEP efficacy estimates, the incidence rate ratio comparing HIV incidence among TGW and MSM, and the number of non-key populations living with HIV. For each of these model inputs, we exponentiate sampled values from a normal distribution with mean ln(m) and standard deviation $\frac{\ln(U) - \ln(L)}{2*1.96}$. Where m is the point estimate, L is the 95% lower confidence limit, and U is the 95% upper confidence limit of the estimate that we have.

We used Beta distributions to sample the HIV prevalence among key populations, the proportion of PWID that are female, and the proportion of FSW that are adherent to PrEP. For each of these inputs we use non-linear least squares (lsqnonlin function in matlab) to fit the parameters of a beta distribution such that its mean and inverse cumulative function at 0.025 and 0.975 correspond to the point estimate, 95% lower confidence limit, and 95% upper confidence limit of the estimate that we have, respectively.

We used Triangular distributions to sample the population size of key populations, HIV incidence, and HIV transmission rates. For each of these model inputs we sample from a triangular distribution with mode m and range $L - U$, where m is the point estimate, L and U are the lower and upper values of the uncertainty range for the estimates that we have.

Supplementary Table 1: Model inputs for key population groups

Country	Group	# on PrEP	HIV Prevalence		Population Size		HIV Incidence	
			Estimate (95%CI)	Source	Estimate (95%CI)	Source	Estimate (95%CI)	Source
Benin	FSW	157	11.7% (8.7 - 15.9)	(2)	35600 (18900 - 73700)	(2)	1.4 (0.8 - 2.5)	Pooled estimate for CWA (3)
Botswana	FSW	248	50.1% (39.8 - 60.8)	(2)	13800 (7400 - 32800)	(2)	3.9 (3.2 - 4.7)	Pooled estimate for ESA (3)
Burkina Faso	FSW	200	9.2% (6.8 - 12.9)	(2)	37000 (19900 - 68600)	(2)	1.4 (0.8 - 2.5)	Pooled estimate for CWA (3)
Burundi	FSW	149	13.2% (8.7 - 20.0)	(2)	35500 (15500 - 75300)	(2)	1.4 (0.8 - 2.5)	Pooled estimate for CWA (3)
Cameroon	FSW	1858	17.4% (11.1 - 25.7)	(2)	120800 (68500 - 223700)	(2)	1.4 (0.8 - 2.5)	Pooled estimate for CWA (3)
Cote d'Ivoire	FSW	462	6.9% (4.6 - 10.2)	(2)	64200 (33600 - 133600)	(2)	1.4 (0.7 - 3.0)	(4)
Democratic Republic of the Congo	FSW	879	8.4% (5.5 - 12.6)	(2)	308800 (189200 - 515600)	(2)	1.4 (0.8 - 2.5)	Pooled estimate for CWA (3)
Eswatini	FSW	201	61.7% (47.0 - 73.9)	(2)	4400 (1800 - 10900)	(2)	3.9 (3.2 - 4.7)	Pooled estimate for ESA (3)
Ethiopia	FSW	13235	14.1% (9.7 - 20.5)	(2)	364700 (168400 - 748100)	(2)	3.9 (3.2 - 4.7)	Pooled estimate for ESA (3)
Ghana	FSW	4	8.0% (5.4 - 11.7)	(2)	32500 (15200 - 66600)	(2)	1.4 (0.8 - 2.5)	Pooled estimate for CWA (3)
Kenya	FSW	12736	25.6% (17.3 - 34.8)	(2)	229300 (121800 - 416400)	(2)	1.8 (1.0 - 3.1)	(5)
Lesotho	FSW	736	57.4% (44.8 - 68.9)	(2)	6700 (2400 - 17600)	(2)	3.9 (3.2 - 4.7)	Pooled estimate for ESA (3)
Liberia	FSW	1479	9.5% (5.6 - 15.6)	(2)	13800 (5100 - 44000)	(2)	1.4 (0.8 - 2.5)	Pooled estimate for CWA (3)
Malawi	FSW	4086	42.6% (30.3 - 54.5)	(2)	70200 (34900 - 135000)	(2)	3.9 (3.2 - 4.7)	Pooled estimate for ESA (3)
Mali	FSW	655	10.4% (7.0 - 15.6)	(2)	40500 (22000 - 77400)	(2)	1.4 (0.8 - 2.5)	Pooled estimate for CWA (3)
Mozambique	FSW	2339	35.1% (26.8 - 44.1)	(2)	97300 (54900 - 170100)	(2)	3.9 (3.2 - 4.7)	Pooled estimate for ESA (3)
Namibia	FSW	625	35.6% (25.8 - 46.4)	(2)	11900 (6400 - 22500)	(2)	3.9 (3.2 - 4.7)	Pooled estimate for ESA (3)
Nigeria	FSW	14742	13.8% (10.3 - 18.3)	(2)	842100 (465800 - 1442500)	(2)	1.4 (0.8 - 2.5)	Pooled estimate for CWA (3)
Rwanda	FSW	10405	27.5% (20.5 - 35.9)	(2)	39100 (15200 - 91500)	(2)	3.9 (3.2 - 4.7)	Pooled estimate for ESA (3)
Senegal	FSW	30	8.0% (4.7 - 13.0)	(2)	34000 (14100 - 70300)	(2)	1.3 (0.5 - 3.5)	(6)
Sierra Leone	FSW	3834	9.6% (5.3 - 16.2)	(2)	23600 (9500 - 61500)	(2)	1.4 (0.8 - 2.5)	Pooled estimate for CWA (3)
South Africa	FSW	2695	58.8% (52.5 - 65.0)	(2)	189600 (95200 - 338000)	(2)	4.6 (1.5 - 8.5)	(7)
South Sudan	FSW	30	21.5% (14.2 - 30.6)	(2)	41400 (20000 - 85600)	(2)	3.9 (3.2 - 4.7)	Pooled estimate for ESA (3)
Tanzania	FSW	43029	27.2% (20.5 - 35.1)	(2)	179900 (104200 - 307400)	(2)	4.7 (2.4 - 9.3)	(8, 9)
Togo	FSW	231	12.6% (9.2 - 16.9)	(2)	24100 (12400 - 49000)	(2)	1.4 (0.8 - 2.5)	Pooled estimate for CWA (3)
Uganda	FSW	3376	33.4% (25.5-42.3)	(2)	141500 (77200 - 257500)	(2)	3.0 (2.4 – 3.9)	(10)
Zambia	FSW	10597	45.0% (37.8 - 53.2)	(2)	71300 (40100 - 116800)	(2)	3.9 (3.2 - 4.7)	Pooled estimate for ESA (3)
Zimbabwe	FSW	7665	48.0% (39.2 - 57.0)	(2)	52900 (25900 - 110000)	(2)	4.3 (3.0 - 6.1)	(11, 12)
Benin	MSM	73	9.6% (6.3 - 14.0)	(2)	20200 (11600 - 36000)	(2)	5.9 (4.5 – 7.9)	(13)
Botswana	MSM	305	16.9% (10.1 - 26.2)	(2)	6100 (3600 - 10100)	(2)	4.7 (2.3 - 11.9)	Estimate for ESA (14)

Country	Group	# on PrEP	HIV Prevalence		Population Size		HIV Incidence	
			Estimate (95%CI)	Source	Estimate (95%CI)	Source	Estimate (95%CI)	Source
Burkina Faso	MSM	24	7.4% (4.7 - 11.4)	(2)	28900 (15800 - 47500)	(2)	7.3 (3.8 - 14.0)	(15)
Burundi	MSM	42	4.1% (2.1 - 8.0)	(2)	18700 (9400 - 36000)	(2)	7.8 (2.8 - 36.4)	Estimate for CWA (14)
Cameroon	MSM	913	18.0% (11.7 - 27.7)	(2)	56600 (33300 - 102400)	(2)	7.8 (2.8 - 36.4)	Estimate for CWA (14)
Cote d'Ivoire	MSM	307	10.4% (7.1 - 14.9)	(2)	42300 (26300 - 68800)	(2)	14.4 (9.6 - 21.7)	(15)
Democratic Republic of the Congo	MSM	229	11.1% (6.1 - 19.9)	(2)	159300 (97800 - 255000)	(2)	7.8 (2.8 - 36.4)	Estimate for CWA (14)
Eswatini	MSM	100	24.4% (15.1 - 37.3)	(2)	2200 (1100 - 4600)	(2)	4.7 (2.3 - 11.9)	Estimate for ESA (14)
Ethiopia	MSM	0	5.4% (1.7 - 15.8)	(2)	183100 (80400 - 381000)	(2)	4.7 (2.3 - 11.9)	Estimate for ESA (14)
Ghana	MSM	1	26.1% (19.3 - 36.0)	(2)	57800 (33800 - 95300)	(2)	7.8 (2.8 - 36.4)	Estimate for CWA (14)
Kenya	MSM	5150	12.1% (8.2 - 18.8)	(2)	82000 (42100 - 143000)	(2)	3.4 (2.2 - 5.3)	(16-19)
Lesotho	MSM	629	29.2% (19.9 - 40.1)	(2)	4400 (1900 - 10100)	(2)	4.7 (2.3 - 11.9)	Estimate for ESA (14)
Liberia	MSM	1268	14.7% (8.7 - 23.9)	(2)	7400 (3900 - 14200)	(2)	7.8 (2.8 - 36.4)	Estimate for CWA (14)
Malawi	MSM	3519	12.7% (9.2 - 17.7)	(2)	33200 (16500 - 61400)	(2)	1.3 (0.2 - 9.5)	(18)
Mali	MSM	738	11.2% (8.0 - 16.0)	(2)	30600 (17600 - 52200)	(2)	9.0 (6.4 - 12.5)	(20)
Mozambique	MSM	1300	13.6% (9.0 - 20.0)	(2)	58300 (34000 - 96200)	(2)	4.7 (2.3 - 11.9)	Estimate for ESA (14)
Namibia	MSM	156	14.4% (8.0 - 24.9)	(2)	5500 (3300 - 10000)	(2)	4.7 (2.3 - 11.9)	Estimate for ESA (14)
Nigeria	MSM	9063	14.0% (9.6 - 19.4)	(2)	381700 (231500 - 651900)	(2)	10.3 (8.1 - 13.1)	(21)
Rwanda	MSM	2174	6.3% (3.5 - 11.0)	(2)	22200 (11500 - 43200)	(2)	4.7 (2.3 - 11.9)	Estimate for ESA (14)
Senegal	MSM	90	22.7% (15.4 - 31.5)	(2)	22300 (10800 - 40700)	(2)	3.2 (1.6 - 6.4)	(6)
Sierra Leone	MSM	270	6.1% (2.8 - 12.7)	(2)	13600 (6400 - 27700)	(2)	7.8 (2.8 - 36.4)	Estimate for CWA (14)
South Africa	MSM	4551	32.9% (27.1 - 39.4)	(2)	125700 (76400 - 197900)	(2)	9.1 (5.7 - 14.7)	(18, 22, 23)
South Sudan	MSM	0	7.5% (3.5 – 16.1)	(2)	13900 (7500 - 27100)	(2)	4.7 (2.3 - 11.9)	Estimate for ESA (14)
Tanzania	MSM	4649	11.8% (8.0 - 17.2)	(2)	108800 (62100 - 178600)	(2)	4.7 (2.3 - 11.9)	Estimate for ESA (14)
Togo	MSM	57	18.4% (12.8 - 25.8)	(2)	14300 (8400 - 24400)	(2)	10.2 (5.7 - 18.5)	(15)
Uganda	MSM	6977	10.5% (5.7 - 16.8)	(2)	75100 (41400 – 130400)	(2)	4.7 (2.3 - 11.9)	Estimate for ESA (14)
Zambia	MSM	7211	14.6% (9.0 - 22.0)	(2)	38100 (22700 - 61600)	(2)	4.7 (2.3 - 11.9)	Estimate for ESA (14)
Zimbabwe	MSM	2752	15.7% (9.4 - 24.8)	(2)	26100 (14400 - 46800)	(2)	4.7 (2.3 - 11.9)	Estimate for ESA (14)
Botswana	PWID	6	25.6% (11.5 - 45.7)	(2)	2000 (900 - 4200)	(2)	3.2 (2.2 - 4.6)	Pooled estimate for LMICs (24)
Burundi	PWID	3	10.2% (5.6 - 16.9)	(25)	6900 (3200 - 14700)	(2)	3.2 (2.2 - 4.6)	Pooled estimate for LMICs (24)
Democratic Republic of the Congo	PWID	3	2.4% (1.7 - 3.2)	(25)	36500 (4500 - 69000)	(25)	3.2 (2.2 - 4.6)	Pooled estimate for LMICs (24)
Eswatini	PWID	22	31.7% (12.8 - 59.7)	(2)	700 (300 - 1600)	(2)	3.2 (2.2 - 4.6)	Pooled estimate for LMICs (24)
Kenya	PWID	153	11.3% (5.0 - 19.8)	(25)	36000 (27500 - 47500)	(25)	2.6 (1.8 - 3.7)	Kenya estimate from (24)

Country	Group	# on PrEP	HIV Prevalence		Population Size		HIV Incidence	
			Estimate (95%CI)	Source	Estimate (95%CI)	Source	Estimate (95%CI)	Source
Lesotho	PWID	2	26.6% (7.5 - 61.0)	(2)	1400 (400 - 3700)	(2)	3.2 (2.2 - 4.6)	Pooled estimate for LMICs (24)
Liberia	PWID	356	5.2% (2.5 - 10.3)	(2)	11000 (1000 - 21000)	(25)	3.2 (2.2 - 4.6)	Pooled estimate for LMICs (24)
Malawi	PWID	38	20.6% (9.0 - 40.1)	(2)	10900 (4800 - 24000)	(2)	3.2 (2.2 - 4.6)	Pooled estimate for LMICs (24)
Mozambique	PWID	192	35.5% (19.5 - 53.4)	(25)	33000 (500 - 68000)	(25)	3.2 (2.2 - 4.6)	Pooled estimate for LMICs (24)
Nigeria	PWID	5093	3.8% (1.4 - 7.4)	(25)	177500 (97500 - 326500)	(25)	3.2 (2.2 - 4.6)	Pooled estimate for LMICs (24)
South Africa	PWID	78	17.9% (14.1 - 22.1)	(25)	82000 (23500 - 288500)	(25)	12.3 (10.9 - 13.8)	(26)
Tanzania	PWID	1329	14.0% (5.0 - 26.5)	(25)	38700 (21400 – 68800)	(2)	3.2 (2.2 - 4.6)	Pooled estimate for LMICs (24)
Uganda	PWID	3376	26.1% (14.2 - 43.7)	(2)	28000 (14800 - 51200)	(2)	3.2 (2.2 - 4.6)	Pooled estimate for LMICs (24)
Zambia	PWID	1617	22.7% (12.1 - 35.4)	(2)	12700 (6500 - 23100)	(2)	3.2 (2.2 - 4.6)	Pooled estimate for LMICs (24)
Zimbabwe	PWID	703	23.0% (10.7 - 40.9)	(2)	9000 (4000 - 18900)	(2)	3.2 (2.2 - 4.6)	Pooled estimate for LMICs (24)
Botswana	TGW	8	25.4% (15.1 - 38.5)	(2)	800 (300 - 2700)	(2)	11.5 (5.1 - 27.5)	Applied IRR to MSM estimate
Burundi	TGW	2	6.5% (3.0 - 14.6)	(2)	2300 (700 - 8300)	(2)	19.5 (6.6 - 68.4)	Applied IRR to MSM estimate
Democratic Republic of the Congo	TGW	2	15.0% (8.0 - 25.9)	(2)	21200 (8400 - 54600)	(2)	19.6 (6.6 - 68.4)	Applied IRR to MSM estimate
Eswatini	TGW	3	37.3% (23.5 - 53.4)	(2)	300 (100 - 1200)	(2)	11.5 (5.1 - 27.5)	Applied IRR to MSM estimate
Kenya	TGW	103	19.6% (12.1 - 30.5)	(2)	10500 (3600 - 32500)	(2)	20.6 (6.6 - 63.9)	(17)
Lesotho	TGW	69	39.4% (27.3 - 53.7)	(2)	600 (100 - 3500)	(2)	11.5 (5.1 - 27.5)	Applied IRR to MSM estimate
Liberia	TGW	66	19.0% (11.3 - 30.8)	(2)	800 (200 - 2400)	(2)	19.6 (6.6 - 68.4)	Applied IRR to MSM estimate
Malawi	TGW	571	19.7% (14.0 - 26.3)	(2)	4100 (1500 - 12000)	(2)	3.1 (0.6 - 17.5)	Applied IRR to MSM estimate
Mozambique	TGW	34	20.6% (13.8 - 29.9)	(2)	7100 (2700 - 17500)	(2)	11.5 (5.1 - 27.5)	Applied IRR to MSM estimate
Namibia	TGW	43	21.3% (11.9 - 34.9)	(2)	700 (300 - 2400)	(2)	11.5 (5.1 - 27.5)	Applied IRR to MSM estimate
Nigeria	TGW	363	18.3% (12.3 - 25.9)	(2)	37100 (12200 - 113800)	(2)	23.9 (13.6 - 39.1)	(21)
Rwanda	TGW	5	9.8% (5.1 - 19.2)	(2)	2800 (900 - 9100)	(2)	11.5 (5.1 - 27.5)	Applied IRR to MSM estimate
South Africa	TGW	407	45.2% (36.3 - 55.1)	(2)	18500 (6900 - 60600)	(2)	31.0 (3.7 - 111.2)	(23)
Uganda	TGW	598	15.8% (8.1 - 27.3)	(2)	9200 (3400 - 27000)	(2)	14.5 (6.3 - 31.2)	Applied IRR to MSM estimate
Zambia	TGW	942	21.3% (13.2 - 31.4)	(2)	4600 (1900 - 13100)	(2)	11.5 (5.1 - 27.5)	Applied IRR to MSM estimate
Zimbabwe	TGW	450	22.4% (13.6 - 33.7)	(2)	3900 (1500 - 10200)	(2)	11.5 (5.1 - 27.5)	Applied IRR to MSM estimate

Supplementary Table 2: Model inputs for non-key population groups.

Country	Age	Population Size (27)		# on PrEP		# Living with HIV (28)		HIV Incidence		
		Female	Male	Female	Male	Female (95%CI)	Male (95%CI)	Female (95%CI)	Male (95%CI)	Source
Benin	15-24	1436846	1478462	46	30	4000 (2900 - 5300)	2700 (2200 - 3200)	0.11 (0.04 - 0.18)	0.04 (0.00 - 0.08)	WCA Estimate (29)
Benin	15-49					30000 (25000 - 37000)	15000 (12000 - 19000)			
Benin	25-34	1060276	1082311	161	121			0.20 (0.08 - 0.31)	0.09 (0.02 - 0.17)	WCA Estimate (29)
Benin	35-49	1050903	1035196	159	116			0.09 (0.01 - 0.18)	0.06 (0.00 - 0.16)	WCA Estimate (29)
Botswana	15-24	244033	245092	369	143	13000 (7100 - 18000)	6500 (4800 - 8200)	0.00 (0.00 - 0.75)	0.00 (0.00 - 0.64)	(30)
Botswana	15-49					160000 (140000 - 160000)	86000 (69000 - 93000)			
Botswana	25-34	242845	242844	987	759			0.44 (0.07 - 0.85)	0.00 (0.00 - 0.69)	(30)
Botswana	35-49	217842	221312	886	691			0.34 (0.02 - 0.75)	0.21 (0.00 - 0.70)	(30)
Burkina Faso	15-24	2495545	2554994	85	15	6000 (4600 - 7300)	4500 (3800 - 5300)	0.11 (0.04 - 0.18)	0.04 (0.00 - 0.08)	WCA Estimate (29)
Burkina Faso	15-49					40000 (34000 - 46000)	23000 (19000 - 27000)			
Burkina Faso	25-34	1731462	1729026	78	24			0.20 (0.08 - 0.31)	0.09 (0.02 - 0.17)	WCA Estimate (29)
Burkina Faso	35-49	1725072	1674381	78	24			0.09 (0.01 - 0.18)	0.06 (0.00 - 0.16)	WCA Estimate (29)
Burundi	15-24	1478935	1477566	138	32	4600 (3100 - 5800)	2900 (2500 - 3500)	0.11 (0.04 - 0.18)	0.04 (0.00 - 0.08)	WCA Estimate (29)
Burundi	15-49					36000 (31000 - 42000)	21000 (17000 - 24000)			
Burundi	25-34	920894	908668	172	98			0.20 (0.08 - 0.31)	0.09 (0.02 - 0.17)	WCA Estimate (29)
Burundi	35-49	1067184	1028788	199	110			0.09 (0.01 - 0.18)	0.06 (0.00 - 0.16)	WCA Estimate (29)
Cameroon	15-24	2971910	2978931	485	388	59500 (44500 - 74500)	12000 (6000 - 21000)	0.64 (0.12 - 1.15)	0.07 (0.00 - 0.21)	(31)
Cameroon	15-49					353500 (309500 - 390500)	146500 (124500 - 168500)			
Cameroon	25-34	2184936	2167236	698	266			0.28 (0.00 - 0.55)	0.13 (0.00 - 0.40)	(31)
Cameroon	35-49	2206796	2169097	705	266			0.22 (0.00 - 0.52)	0.04 (0.00 - 0.17)	(31)
Côte_d'Ivoire	15-24	3120924	3325178	2120	349	24000 (16000 - 31000)	13000 (10000 - 16000)	0.04 (0.00 - 0.15)	0.09 (0.00 - 0.27)	(31)
Côte_d'Ivoire	15-49					200000 (170000 - 230000)	83000 (70000 - 97000)			
Côte_d'Ivoire	25-34	2265355	2285865	425	739			0.05 (0.00 - 0.20)	0.00 (0.00 - 0.43)	(31)
Côte_d'Ivoire	35-49	2614269	2664661	490	861			0.00 (0.00 - 0.51)	0.00 (0.00 - 0.43)	(31)
Democratic Republic of the Congo	15-24	10817120	10741738	595	273	31000 (19000 - 46000)	19000 (14000 - 26000)	0.11 (0.04 - 0.18)	0.04 (0.00 - 0.08)	WCA Estimate (29)
Democratic Republic of the Congo	15-49					230000 (180000 - 290000)	130000 (98000 - 170000)			
Democratic Republic of the Congo	25-34	7606217	7485641	939	791			0.20 (0.08 - 0.31)	0.09 (0.02 - 0.17)	WCA Estimate (29)

Country	Age	Population Size (27)		# on PrEP		# Living with HIV (28)		HIV Incidence		
		Female	Male	Female	Male	Female (95%CI)	Male (95%CI)	Female (95%CI)	Male (95%CI)	Source
Democratic Republic of the Congo	35-49	6823549	6630515	842	700			0.09 (0.01 - 0.18)	0.06 (0.00 - 0.16)	WCA Estimate (29)
Eswatini	15-24	124305	125262	2935	439	9000 (5200 - 11000)	5500 (3500 - 7200)	1.63 (0.43 - 2.81)	0.17 (0.00 - 0.50)	(31)
Eswatini	15-49					99000 (96000 - 100000)	65000 (61000 - 68000)			
Eswatini	25-34	103810	104636	1102	568			1.90 (0.45 - 3.33)	0.38 (0.00 - 1.13)	(31)
Eswatini	35-49	113639	111171	1207	604			0.34 (0.02 - 0.75)	0.21 (0.00 - 0.70)	(31)
Ethiopia	15-24	14065346	14362372	7286	83	33000 (23000 - 48000)	22000 (17000 - 28000)	0.00 (0.00 - 0.22)	0.03 (0.00 - 0.14)	(31)
Ethiopia	15-49					260000 (210000 - 330000)	130000 (100000 - 170000)			
Ethiopia	25-34	10659597	10750422	3387	195			0.09 (0.00 - 0.25)	0.00 (0.00 - 0.46)	(31)
Ethiopia	35-49	9332597	9249690	2965	167			0.13 (0.00 - 0.38)	0.00 (0.00 - 0.63)	(31)
Ghana	15-24	3352714	3400342	35	17	22000 (12000 - 30000)	11000 (6800 - 16000)	0.11 (0.04 - 0.18)	0.04 (0.00 - 0.08)	WCA Estimate (29)
Ghana	15-49					170000 (140000 - 200000)	87000 (71000 - 100000)			
Ghana	25-34	2625436	2639795	27	28			0.20 (0.08 - 0.31)	0.09 (0.02 - 0.17)	WCA Estimate (29)
Ghana	35-49	3012492	2987044	30	31			0.09 (0.01 - 0.18)	0.06 (0.00 - 0.16)	WCA Estimate (29)
Kenya	15-24	6215090	6163392	12961	5758	84000 (63000 - 100000)	53000 (45000 - 61000)	0.00 (0.00 - 0.22)	0.14 (0.00 - 0.33)	(31)
Kenya	15-49					670000 (600000 - 760000)	320000 (270000 - 370000)			
Kenya	25-34	4589867	4374464	14472	9667			0.28 (0.00 - 0.69)	0.16 (0.00 - 0.41)	(31)
Kenya	35-49	4515163	4600826	14238	10168			0.24 (0.00 - 0.53)	0.15 (0.00 - 0.45)	(31)
Lesotho	15-24	234556	234601	2145	658	13000 (8000 - 17000)	6000 (4000 - 7700)	0.34 (0.00 - 0.74)	0.33 (0.00 - 0.78)	(31)
Lesotho	15-49					120000 (120000 - 130000)	70000 (61000 - 75000)			
Lesotho	25-34	203054	197445	1583	1459			1.55 (0.44 - 2.64)	0.55 (0.00 - 1.23)	(31)
Lesotho	35-49	224351	208734	1749	1543			0.72 (0.00 - 1.55)	0.00 (0.00 - 1.02)	(31)
Liberia	15-24	596100	607261	1591	1372	2800 (1800 - 4000)	1600 (1300 - 2100)	0.11 (0.04 - 0.18)	0.04 (0.00 - 0.08)	WCA Estimate (29)
Liberia	15-49					16000 (13000 - 20000)	8900 (7400 - 11000)			
Liberia	25-34	404386	404748	541	689			0.20 (0.08 - 0.31)	0.09 (0.02 - 0.17)	WCA Estimate (29)
Liberia	35-49	425181	415772	568	708			0.09 (0.01 - 0.18)	0.06 (0.00 - 0.16)	WCA Estimate (29)
Malawi	15-24	2440092	2396060	4255	2400	56000 (42000 - 65000)	34000 (29000 - 38000)	0.42 (0.09 - 0.75)	0.07 (0.00 - 0.22)	(31)
Malawi	15-49					460000 (440000 - 490000)	250000 (220000 - 270000)			
Malawi	25-34	1729287	1636183	2687	2447			0.25 (0.00 - 0.55)	0.38 (0.00 - 0.82)	(31)
Malawi	35-49	1707881	1528997	2654	2286			0.18 (0.00 - 0.45)	0.00 (0.00 - 0.51)	(31)
Mali	15-24	2559841	2625948	303	394	12000 (8500 - 16000)	5700 (4100 - 8000)	0.11 (0.04 - 0.18)	0.04 (0.00 - 0.08)	WCA Estimate (29)

Country	Age	Population Size (27)		# on PrEP		# Living with HIV (28)		HIV Incidence		
		Female	Male	Female	Male	Female (95%CI)	Male (95%CI)	Female (95%CI)	Male (95%CI)	Source
Mali	15-49					58000 (47000 - 74000)	32000 (25000 - 41000)			
Mali	25-34	1608813	1653393	178	174			0.20 (0.08 - 0.31)	0.09 (0.02 - 0.17)	WCA Estimate (29)
Mali	35-49	1570236	1616678	174	170			0.09 (0.01 - 0.18)	0.06 (0.00 - 0.16)	WCA Estimate (29)
Mozambique	15-24	3617627	3589702	6201	3240	210000 (110000 - 290000)	83000 (41000 - 120000)	0.72 (0.14 - 1.29)	0.28 (0.00 - 0.69)	(31)
Mozambique	15-49					1200000 (1100000 - 1300000)	670000 (600000 - 720000)			
Mozambique	25-34	2617117	2494932	3781	3389			0.68 (0.00 - 1.40)	0.15 (0.00 - 0.51)	(31)
Mozambique	35-49	2427769	2079130	3508	2824			0.76 (0.00 - 1.75)	0.21 (0.00 - 0.63)	(31)
Namibia	15-24	274097	275522	2461	109	14000 (9000 - 17000)	6100 (4600 - 7300)	0.99 (0.30 - 1.68)	0.03 (0.00 - 0.14)	(31)
Namibia	15-49					100000 (94000 - 110000)	50000 (44000 - 55000)			
Namibia	25-34	259075	251970	2161	1238			0.30 (0.00 - 0.69)	0.00 (0.00 - 0.62)	(31)
Namibia	35-49	264373	251780	2206	1237			0.56 (0.00 - 1.41)	0.53 (0.00 - 1.13)	(31)
Nigeria	15-24	24361014	25295784	9366	6020	100000 (58000 - 130000)	60000 (42000 - 77000)	0.05 (0.01 - 0.10)	0.03 (0.00 - 0.07)	(32)
Nigeria	15-49					920000 (850000 - 990000)	510000 (440000 - 560000)			
Nigeria	25-34	16420743	16998814	6605	8122			0.22 (0.08 - 0.37)	0.10 (0.01 - 0.19)	(32)
Nigeria	35-49	16611795	17018480	6682	8131			0.10 (0.02 - 0.18)	0.05 (0.00 - 0.15)	(32)
Rwanda	15-24	1522200	1499148	4994	704	12000 (9200 - 13000)	7800 (7000 - 8600)	0.06 (0.00 - 0.20)	0.05 (0.00 - 0.15)	(31)
Rwanda	15-49					100000 (95000 - 110000)	53000 (45000 - 58000)			
Rwanda	25-34	1081767	1044452	3761	762			0.04 (0.00 - 0.14)	0.24 (0.00 - 0.51)	(31)
Rwanda	35-49	1227937	1139803	4270	831			0.08 (0.00 - 0.22)	0.00 (0.00 - 0.33)	(31)
Senegal	15-24	1903734	2005524	2	27	1800 (1600 - 2300)	1400 (1000 - 1800)	0.11 (0.04 - 0.18)	0.04 (0.00 - 0.08)	WCA Estimate (29)
Senegal	15-49					16000 (14000 - 19000)	12000 (11000 - 15000)			
Senegal	25-34	1473874	1468318	14	32			0.20 (0.08 - 0.31)	0.09 (0.02 - 0.17)	WCA Estimate (29)
Senegal	35-49	1401929	1385025	14	31			0.09 (0.01 - 0.18)	0.06 (0.00 - 0.16)	WCA Estimate (29)
Sierra Leone	15-24	903508	914451	1912	162	9100 (4900 - 13000)	3800 (2100 - 5500)	0.11 (0.04 - 0.18)	0.04 (0.00 - 0.08)	WCA Estimate (29)
Sierra Leone	15-49					40000 (33000 - 48000)	21000 (17000 - 25000)			
Sierra Leone	25-34	683419	684883	976	55			0.20 (0.08 - 0.31)	0.09 (0.02 - 0.17)	WCA Estimate (29)
Sierra Leone	35-49	662888	661087	946	53			0.09 (0.01 - 0.18)	0.06 (0.00 - 0.16)	WCA Estimate (29)
South Africa	15-24	5239444	5329131	51370	5913	430000 (210000 - 620000)	190000 (120000 - 260000)	1.51 (1.31 - 1.71)	0.49 (0.27 - 0.71)	(33)
South Africa	15-49					3900000 (3300000 - 4500000)	2000000 (1500000 - 2200000)			
South Africa	25-34	5638288	5560224	26372	11616			0.45 (0.39 - 0.51)	0.54 (0.48 - 0.60)	(33)
South Africa	35-49	7087879	6966881	33152	14554			0.45 (0.39 - 0.51)	0.54 (0.48 - 0.60)	(33)

Country	Age	Population Size (27)		# on PrEP		# Living with HIV (28)		HIV Incidence		
		Female	Male	Female	Male	Female (95%CI)	Male (95%CI)	Female (95%CI)	Male (95%CI)	Source
South Sudan	15-24	1413834	1431090	29	15	12000 (6600 - 17000)	6300 (3400 - 9000)	0.18 (0.08 - 0.27)	0.10 (0.01 - 0.20)	EA Estimate (29)
South Sudan	15-49					71000 (57000 - 85000)	39000 (31000 - 48000)			
South Sudan	25-34	748833	736951	20	26			0.45 (0.24 - 0.66)	0.18 (0.07 - 0.30)	EA Estimate (29)
South Sudan	35-49	933289	829711	26	29			0.18 (0.07 - 0.30)	0.30 (0.10 - 0.50)	EA Estimate (29)
Tanzania	15-24	7083788	7126087	29285	5748	91000 (49000 - 120000)	47000 (31000 - 62000)	0.33 (0.09 - 0.58)	0.00 (0.00 - 0.25)	(31)
Tanzania	15-49					800000 (760000 - 840000)	370000 (330000 - 400000)			
Tanzania	25-34	5089472	5075722	21175	10101			0.32 (0.04 - 0.59)	0.22 (0.00 - 0.49)	(31)
Tanzania	35-49	4947468	4676598	20585	9307			0.20 (0.00 - 0.43)	0.19 (0.00 - 0.44)	(31)
Togo	15-24	943576	967324	79	45	6100 (4200 - 8200)	3600 (2800 - 4400)	0.11 (0.04 - 0.18)	0.04 (0.00 - 0.08)	WCA Estimate (29)
Togo	15-49					49000 (41000 - 59000)	24000 (19000 - 30000)			
Togo	25-34	686497	705652	120	97			0.20 (0.08 - 0.31)	0.09 (0.02 - 0.17)	WCA Estimate (29)
Togo	35-49	734625	751848	128	104			0.09 (0.01 - 0.18)	0.06 (0.00 - 0.16)	WCA Estimate (29)
Uganda	15-24	5530361	5512823	42002	17493	110000 (64000 - 130000)	44000 (30000 - 57000)	0.62 (0.28 - 0.95)	0.00 (0.00 - 0.23)	(31)
Uganda	15-49					740000 (700000 - 790000)	380000 (330000 - 420000)			
Uganda	25-34	4012358	3948743	25434	23426			0.32 (0.03 - 0.62)	0.47 (0.05 - 0.89)	(31)
Uganda	35-49	3171182	3055074	20102	18124			0.16 (0.00 - 0.38)	0.33 (0.00 - 0.69)	(31)
Zambia	15-24	2284458	2260994	17207	5869	79000 (41000 - 110000)	31000 (20000 - 41000)	0.94 (0.44 - 1.44)	0.08 (0.00 - 0.25)	(31)
Zambia	15-49					640000 (610000 - 680000)	350000 (310000 - 370000)			
Zambia	25-34	1653652	1626137	9061	9498			1.07 (0.40 - 1.73)	0.25 (0.00 - 0.62)	(31)
Zambia	35-49	1633234	1625559	8949	9495			1.06 (0.30 - 1.81)	0.73 (0.05 - 1.40)	(31)
Zimbabwe	15-24	1816261	1798406	11589	1809	64000 (47000 - 77000)	44000 (36000 - 50000)	0.76 (0.25 - 1.26)	0.08 (0.00 - 0.27)	(31)
Zimbabwe	15-49					580000 (540000 - 640000)	350000 (310000 - 380000)			
Zimbabwe	25-34	1160195	1000895	6997	3531			0.65 (0.06 - 1.24)	0.30 (0.00 - 0.77)	(31)
Zimbabwe	35-49	1536711	1206028	9269	4255			0.53 (0.00 - 1.06)	0.41 (0.00 - 1.00)	(31)

Supplementary Table 3: Model inputs - proportion of PWID that are female

Country	Proportion of PWID that are female (95%CI)	Source
Benin	2.9% (1.6 - 4.6)	(25)
Botswana	10.8% (7.2 - 15.2)	Regional estimate (25)
Burkina Faso	10.8% (7.2 - 15.2)	Regional estimate (25)
Burundi	45.0% (41.4 - 48.6)	(25)
Cameroon	0.0% (0.0 - 3.5)	(25)
Cote d'Ivoire	7.0% (1.9 - 17.0)	(25)
Democratic Republic of the Congo	10.8% (7.2 - 15.2)	Regional estimate (25)
Eswatini	10.8% (7.2 - 15.2)	Regional estimate (25)
Ethiopia	6.1% (4.4 - 8.1)	(25)
Ghana	9.1% (6.1 - 12.7)	(25)
Kenya	11.7% (9.0 -14.7)	(25)
Lesotho	10.8% (7.2 - 15.2)	Regional estimate (25)
Liberia	5.6% (0.1 - 17.6)	(25)
Malawi	10.8% (7.2 - 15.2)	Regional estimate (25)
Mali	10.8% (7.2 - 15.2)	Regional estimate (25)
Mozambique	5.0% (3.2 - 7.1)	(25)
Namibia	10.8% (7.2 - 15.2)	Regional estimate (25)
Nigeria	11.9% (8.7 - 15.4)	(25)
Rwanda	18.2% (14.1 - 23.0)	(25)
Senegal	10.8% (7.2 - 15.2)	Regional estimate (25)
Sierra Leone	7.3% (4.5 - 11.2)	(25)
South Africa	15.6% (12.4 - 19.0)	(25)
South Sudan	10.8% (7.2 - 15.2)	Regional estimate (25)
Uganda	18.4% (12.0 - 26.3)	(25)
Tanzania	10.8% (6.5 - 16.1)	(25)
Togo	9.9% (7.2 - 12.9)	(25)
Zambia	10.8% (7.2 - 15.2)	Regional estimate (25)
Zimbabwe	10.8% (7.2 - 15.2)	Regional estimate (25)

Supplementary Table 4: Model inputs – effectiveness of PrEP

Subgroup	Estimate (95%CI)	Source
MSM and TGW	75.0% (95%CI: 39.0-90.0)	(34)
FSW	38.8% (95%UI: 27.0-51.4)	Pooled estimates of the proportion of FSW with high levels of adherence (based on Plasma drug-level testing) to PrEP after 3 months (49%, 95%CI: 32-66) from (35-39). To these FSW, we applied an estimated efficacy of PrEP for highly adherent populations using data from a recent systematic review(40) (79.9%; 95% CI: 67.2–87.6; details in (41)), while we assumed no efficacy for FSW with undetectable drug levels/low adherence.
PWID	48.9% (95%CI: 9.6-72.2)	(42)
Non-key population Men	69% (95%CI: 56-78)	Pooled estimates from (43, 44)
Non-key population Women	31% (95%CI: -10-57%)	Pooled estimates from (43-47). Note, we assume no detrimental effect of PrEP by replacing sampled negative efficacy values with 0.

Supplementary Table 5: Model inputs – HIV transmission rates for different subgroups and sub-regions of sub-Saharan Africa.
Numbers in brackets are range across different models and the number of models 'n' used.

Subgroup	Transmission rate per 100 years of living with HIV* (Uncertainty Range)	
	ESA	WCA
FSW	13.7 (3.7 – 33.1; n=10)	14.6 (10.4 – 21.8; n=5)
MSM	3.8 (0.3 – 13.7; n=8)	8.9 (5.7 – 23.3; n=5)
Non-key population Females 15-24	5.4 (1.7 – 23.2; n=8)	5.7 (4.0 – 8.0; n=4)
Non-key population Females 25-34<br>Non-key population Females 35-49	1.6 (1.1 – 3.4; n=6)	3.3 (1.4 – 3.8; n=4)
Non-key population Males 15-24	3.2 (0.5 – 10.6; n=8)	3.6 (0.2 -8.7; n=4)
Non-key population Males 25-34<br>Non-key population Males 35-49	3.0 (2.3 – 6.1; n=6)	6.8 (4.1 – 9.4; n=4)

* These rates were calculated for a model comparison of HIV acquisition and transmission indicators in Africa (MedRxiv: https://doi.org/10.1101/2025.03.17.25324139). In each of the 15 models, we used the estimated total number of new infections in 2020 $'Inf_{2020}'$ from the model, and the estimated number of new infections in 2020 that would have occurred if subgroup *i* could not transmit HIV $'Inf^{i}_{2020}'$ (obtained using a counterfactual scenario), to calculate the total number of excess infections occurring when subgroup *i* can transmit HIV over the year 2020 $(Inf_{2020} - Inf^{i}_{2020})$. The HIV transmission rate $'Tr^{i}_{2020}'$ (Equation 1) (expressed per 100 years of living with HIV) from subgroup *i* was obtained by dividing the number of excess infections when subgroup *i* can transmit by the cumulative number of person-years lived in the infected subgroup *i* over 2020 $(PLHIV(i)_{2020})$.

$$Tr^{i}_{2020} = \left(\frac{Inf_{2020} - Inf^{i}_{2020}}{PLHIV(i)_{2020}}\right) \times 100 \qquad \text{Equation 1}$$

Supplementary Table 6: Baseline results - PrEP coverage among HIV-negatives

Cells present median projection with 95% uncertainty interval in parentheses, given to 1 significant figure after the decimal point. * No PEPFAR funded PrEP.

Country	FSW	MSM	PWID	TGW	Females 15-24	Females 25-34	Females 35-49	Males 15-24	Males 25-34	Males 35-49
Benin	0.4% (0.3 - 0.8)	0.4% (0.2 - 0.6)	0*	0*	0.002% (0.002 - 0.002)	0.005% (0.005 - 0.005)	0.005% (0.005 - 0.005)	0.001% (0.001 - 0.001)	0.005% (0.005 - 0.005)	0.005% (0.005 - 0.005)
Botswana	2.9% (1.6 - 5.5)	5.7% (3.9 - 8.8)	0.4% (0.2 - 0.7)	0.9% (0.4 - 2.3)	0.1% (0.1 - 0.1)	0.3% (0.3 - 0.3)	0.3% (0.3 - 0.3)	0.05% (0.05 - 0.05)	0.2% (0.2 - 0.2)	0.2% (0.2 - 0.2)
Burkina Faso	0.5% (0.4 - 0.9)	0.1% (0.1 - 0.1)	0*	0*	0.0006% (0.0006 - 0.0006)	0.0005% (0.0005 - 0.0005)	0.0005% (0.0005 - 0.0005)	0.0004% (0.0004 - 0.0004)	0.0005% (0.0005 - 0.0005)	0.0005% (0.0005 - 0.0005)
Burundi	0.4% (0.3 - 0.8)	0.2% (0.1 - 0.4)	0.04% (0.03 - 0.1)	0.1% (0.03 - 0.2)	0.007% (0.007 - 0.007)	0.008% (0.008 - 0.008)	0.008% (0.008 - 0.008)	0.002% (0.002 - 0.002)	0.005% (0.005 - 0.005)	0.005% (0.005 - 0.005)
Cameroon	1.7% (1.1 - 2.8)	1.8% (1.2 - 2.9)	0*	0*	0.0003% (0.0003 - 0.0003)	0.0003% (0.0003 - 0.0003)	0.0003% (0.0003 - 0.0003)	0.0001% (0.0001 - 0.0001)	6e-05% (6e-05 - 6e-05)	6e-05% (6e-05 - 6e-05)
Cote d'Ivoire	0.7% (0.4 - 1.2)	0.8% (0.5 - 1.1)	0*	0*	0.1% (0.1 - 0.1)	0.01% (0.01 - 0.01)	0.01% (0.01 - 0.01)	0.009% (0.009 - 0.009)	0.02% (0.02 - 0.02)	0.02% (0.02 - 0.02)
Democratic Republic of the Congo	0.3% (0.2 - 0.4)	0.2% (0.1 - 0.2)	0.008% (0.005 - 0.03)	0.009% (0.005 - 0.02)	0.004% (0.004 - 0.004)	0.005% (0.005 - 0.005)	0.005% (0.005 - 0.005)	0.002% (0.002 - 0.002)	0.005% (0.005 - 0.005)	0.005% (0.005 - 0.005)
Eswatini	9.6% (4.8 - 22.2)	5.2% (3.2 - 9.7)	4.0% (2.1 - 9.0)	1.0% (0.4 - 2.9)	2.5% (2.4 - 2.5)	1.0% (1.0 - 1.0)	1.0% (1.0 - 1.0)	0.3% (0.3 - 0.3)	0.4% (0.4 - 0.4)	0.4% (0.4 - 0.4)
Ethiopia	3.7% (2.3 - 7.0)	0*	0*	0*	0.002% (0.002 - 0.002)	0.0006% (0.0006 - 0.0006)	0.0006% (0.0006 - 0.0006)	0.0006% (0.0006 - 0.0006)	0.001% (0.001 - 0.001)	0.001% (0.001 - 0.001)
Ghana	0.01% (0.007 - 0.02)	0.002% (0.002 - 0.003)	0*	0*	0.001% (0.001 - 0.001)	0.0006% (0.0006 - 0.0006)	0.0006% (0.0006 - 0.0006)	0.0005% (0.0005 - 0.0005)	0.0007% (0.0007 - 0.0007)	0.0007% (0.0006 - 0.0007)
Kenya	6.8% (4.4 - 11.5)	6.7% (4.5 - 11.4)	0.5% (0.4 - 0.6)	0.9% (0.4 - 2.2)	0.1% (0.1 - 0.2)	0.1% (0.1 - 0.1)	0.1% (0.1 - 0.1)	0.1% (0.1 - 0.1)	0.1% (0.1 - 0.1)	0.1% (0.1 - 0.1)
Lesotho	20.4% (10.4 - 49.3)	16.9% (9.7 - 34.8)	0.2% (0.1 - 0.5)	9.0% (3.7 - 38.5)	0.8% (0.8 - 0.9)	0.6% (0.5 - 0.6)	0.6% (0.5 - 0.6)	0.2% (0.2 - 0.2)	0.4% (0.4 - 0.4)	0.4% (0.4 - 0.4)
Liberia	8.3% (4.2 - 20.6)	18.1% (11.5 - 31.0)	3.4% (2.0 - 11.5)	7.6% (3.9 - 21.7)	0.1% (0.1 - 0.1)	0.04% (0.04 - 0.04)	0.04% (0.04 - 0.04)	0.1% (0.1 - 0.1)	0.04% (0.04 - 0.04)	0.04% (0.04 - 0.04)
Malawi	9.1% (5.5 - 16.6)	11.2% (7.2 - 19.4)	0.4% (0.2 - 0.8)	12.8% (6.7 - 30.7)	0.1% (0.1 - 0.1)	0.1% (0.1 - 0.1)	0.1% (0.1 - 0.1)	0.04% (0.04 - 0.04)	0.04% (0.04 - 0.04)	0.04% (0.04 - 0.04)
Mali	1.6% (1.0 - 2.7)	2.5% (1.7 - 4.0)	0*	0*	0*	0*	0*	0*	0*	0*
Mozambique	3.4% (2.3 - 5.6)	2.4% (1.7 - 3.8)	0.9% (0.5 - 4.0)	0.5% (0.3 - 1.1)	0.2% (0.1 - 0.2)	0.1% (0.1 - 0.1)	0.1% (0.1 - 0.1)	0.1% (0.1 - 0.1)	0.1% (0.1 - 0.1)	0.1% (0.1 - 0.1)
Namibia	7.4% (4.6 - 12.7)	3.0% (2.0 - 4.8)	0*	5.2% (2.6 - 12.6)	0.9% (0.9 - 0.9)	0.6% (0.6 - 0.6)	0.6% (0.6 - 0.6)	0.04% (0.04 - 0.04)	0.3% (0.3 - 0.3)	0.3% (0.3 - 0.3)
Nigeria	1.9% (1.3 - 3.0)	2.6% (1.8 - 3.9)	2.7% (1.8 - 4.5)	0.9% (0.4 - 2.2)	0.01% (0.01 - 0.01)	0.008% (0.008 - 0.008)	0.008% (0.008 - 0.008)	0.008% (0.008 - 0.008)	0.01% (0.01 - 0.01)	0.01% (0.01 - 0.01)
Rwanda	30.8% (17.5 - 65.9)	9.3% (5.9 - 16.2)	0*	0.1% (0.1 - 0.4)	0.1% (0.1 - 0.1)	0.04% (0.04 - 0.04)	0.04% (0.04 - 0.04)	0.002% (0.002 - 0.002)	0.002% (0.002 - 0.002)	0.002% (0.002 - 0.002)
Senegal	0.1% (0.1 - 0.2)	0.5% (0.3 - 0.9)	0*	0*	0*	0*	0*	0*	0*	0*
Sierra Leone	14.1% (7.8 - 31.0)	1.9% (1.2 - 3.5)	0*	0*	0*	0*	0*	0*	0*	0*
South Africa	3.2% (2.1 - 5.6)	5.2% (3.7 - 7.8)	0.1% (0.04 - 0.2)	2.8% (1.4 - 7.0)	1.1% (1.0 - 1.1)	0.4% (0.4 - 0.5)	0.4% (0.4 - 0.5)	0.1% (0.1 - 0.1)	0.1% (0.1 - 0.1)	0.1% (0.1 - 0.1)
South Sudan	0.1% (0.05 - 0.1)	0*	0*	0*	0.001% (0.001 - 0.001)	0.0009% (0.0009 - 0.0009)	0.0009% (0.0009 - 0.001)	0.001% (0.001 - 0.001)	0.002% (0.002 - 0.002)	0.002% (0.002 - 0.002)
Tanzania	30.6% (20.7 - 48.4)	4.6% (3.2 - 7.2)	3.7% (2.4 - 6.1)	0*	0.2% (0.2 - 0.2)	0.1% (0.1 - 0.1)	0.1% (0.1 - 0.1)	0.1% (0.1 - 0.1)	0.1% (0.1 - 0.1)	0.1% (0.1 - 0.1)

Country	FSW	MSM	PWID	TGW	Females 15-24	Females 25-34	Females 35-49	Males 15-24	Males 25-34	Males 35-49
Togo	1.0% (0.6 - 1.7)	0.5% (0.3 - 0.7)	0*	0*	0.003% (0.002 - 0.003)	0.003% (0.003 - 0.003)	0.003% (0.003 - 0.003)	0.004% (0.004 - 0.004)	0.006% (0.006 - 0.007)	0.007% (0.007 - 0.007)
Uganda	3.3% (2.1 - 5.5)	9.7% (6.5 - 15.7)	15.2% (9.5 - 26.5)	5.7% (3.0 - 13.7)	0.8% (0.7 - 0.8)	0.4% (0.4 - 0.4)	0.4% (0.4 - 0.4)	0.3% (0.3 - 0.3)	0.3% (0.3 - 0.3)	0.3% (0.3 - 0.3)
Zambia	25.8% (17.5 - 41.4)	21.0% (14.8 - 32.1)	15.2% (9.7 - 26.4)	19.4% (10.3 - 43.7)	0.6% (0.5 - 0.6)	0.3% (0.3 - 0.3)	0.3% (0.3 - 0.3)	0.2% (0.2 - 0.2)	0.2% (0.2 - 0.2)	0.2% (0.2 - 0.2)
Zimbabwe	24.3% (14.4 - 45.3)	11.5% (7.6 - 18.9)	8.9% (5.2 - 17.8)	11.8% (6.3 - 26.5)	0.5% (0.5 - 0.5)	0.3% (0.3 - 0.3)	0.3% (0.3 - 0.3)	0.1% (0.1 - 0.1)	0.1% (0.1 - 0.1)	0.1% (0.1 - 0.1)

Supplementary Table 7: Baseline results - Relative impact of removing PEPFAR funded PrEP for 1-year (Primary Infections)

Cells present median projection with 95% uncertainty interval in parentheses, given to 1 significant figure after the decimal point. * No PEPFAR funded PrEP.

Country	FSW	MSM	PWID	TGW	Females 15-24	Females 25-34	Females 35-49	Males 15-24	Males 25-34	Males 35-49
Benin	0.2% (0.1 - 0.3)	0.3% (0.2 - 0.5)	0*	0*	0.0006% (0 - 0.001)	0.002% (0 - 0.003)	0.002% (0 - 0.003)	0.001% (0.0009 - 0.001)	0.003% (0.003 - 0.004)	0.003% (0.003 - 0.004)
Botswana	1.1% (0.5 - 2.4)	4.3% (2.4 - 7.4)	0.2% (0.1 - 0.4)	0.7% (0.3 - 1.8)	0.05% (0 - 0.1)	0.1% (0 - 0.2)	0.1% (0 - 0.2)	0.03% (0.03 - 0.04)	0.1% (0.1 - 0.1)	0.1% (0.1 - 0.1)
Burkina Faso	0.2% (0.1 - 0.4)	0.1% (0.04 - 0.1)	0*	0*	0.0002% (0 - 0.0003)	0.0001% (0 - 0.0002)	0.0001% (0 - 0.0002)	0.0003% (0.0002 - 0.0003)	0.0003% (0.0003 - 0.0004)	0.0004% (0.0003 - 0.0004)
Burundi	0.2% (0.1 - 0.3)	0.2% (0.1 - 0.3)	0.02% (0.006 - 0.04)	0.04% (0.02 - 0.1)	0.002% (0 - 0.004)	0.002% (0 - 0.004)	0.002% (0 - 0.004)	0.001% (0.001 - 0.001)	0.003% (0.003 - 0.004)	0.003% (0.003 - 0.004)
Cameroon	0.6% (0.4 - 1.2)	1.3% (0.7 - 2.3)	0*	0*	8e-05% (0 - 0.0001)	0.0001% (0 - 0.0002)	0.0001% (0 - 0.0002)	7e-05% (6e-05 - 8e-05)	4e-05% (3e-05 - 4e-05)	4e-05% (3e-05 - 4e-05)
Cote d'Ivoire	0.3% (0.1 - 0.5)	0.6% (0.3 - 0.9)	0*	0*	0.02% (0 - 0.03)	0.003% (0 - 0.005)	0.003% (0 - 0.005)	0.006% (0.005 - 0.007)	0.01% (0.01 - 0.01)	0.01% (0.01 - 0.01)
Democratic Republic of the Congo	0.1% (0.1 - 0.2)	0.1% (0.1 - 0.2)	0.004% (0.001 - 0.01)	0.006% (0.003 - 0.01)	0.001% (0 - 0.002)	0.001% (0 - 0.002)	0.001% (0 - 0.002)	0.002% (0.001 - 0.002)	0.004% (0.003 - 0.004)	0.004% (0.003 - 0.004)
Eswatini	3.8% (1.7 - 10.1)	3.9% (2.0 - 8.0)	1.9% (0.6 - 5.0)	0.7% (0.3 - 2.2)	0.8% (0 - 1.4)	0.3% (0 - 0.5)	0.3% (0 - 0.5)	0.2% (0.2 - 0.3)	0.3% (0.2 - 0.3)	0.3% (0.2 - 0.3)
Ethiopia	1.5% (0.7 - 3.0)	0*	0*	0*	0.0005% (0 - 0.0008)	0.0002% (0 - 0.0003)	0.0002% (0 - 0.0003)	0.0004% (0.0003 - 0.0004)	0.0007% (0.0006 - 0.0008)	0.0007% (0.0006 - 0.0008)
Ghana	0.005% (0.002 - 0.009)	0.002% (0.0009 - 0.003)	0*	0*	0.0003% (0 - 0.0005)	0.0002% (0 - 0.0003)	0.0002% (0 - 0.0003)	0.0003% (0.0003 - 0.0004)	0.0005% (0.0004 - 0.0005)	0.0004% (0.0004 - 0.0005)
Kenya	2.7% (1.5 - 5.1)	5.2% (2.8 - 9.6)	0.2% (0.1 - 0.4)	0.6% (0.3 - 1.7)	0.05% (0 - 0.1)	0.04% (0 - 0.1)	0.04% (0 - 0.1)	0.1% (0.04 - 0.1)	0.1% (0.1 - 0.1)	0.1% (0.1 - 0.1)
Lesotho	8.5% (3.6 - 25.3)	14.0% (6.6 - 35.6)	0.1% (0.02 - 0.2)	7.0% (2.5 - 40.0)	0.3% (0 - 0.5)	0.2% (0 - 0.3)	0.2% (0 - 0.3)	0.2% (0.1 - 0.2)	0.3% (0.3 - 0.3)	0.3% (0.3 - 0.3)
Liberia	3.3% (1.4 - 9.2)	15.1% (7.7 - 31.3)	1.7% (0.5 - 6.2)	5.9% (2.6 - 19.3)	0.04% (0 - 0.1)	0.01% (0 - 0.02)	0.01% (0 - 0.02)	0.1% (0.1 - 0.1)	0.03% (0.02 - 0.03)	0.03% (0.02 - 0.03)
Malawi	3.6% (1.8 - 7.5)	8.8% (4.7 - 17.6)	0.2% (0.1 - 0.4)	10.2% (4.5 - 30.0)	0.03% (0 - 0.1)	0.02% (0 - 0.03)	0.02% (0 - 0.03)	0.03% (0.03 - 0.03)	0.03% (0.02 - 0.03)	0.03% (0.02 - 0.03)
Mali	0.6% (0.3 - 1.2)	1.9% (1.1 - 3.2)	0*	0*	0*	0*	0*	0*	0*	0*
Mozambique	1.3% (0.7 - 2.4)	1.8% (1.0 - 3.0)	0.4% (0.1 - 2.0)	0.4% (0.2 - 0.8)	0.05% (0 - 0.1)	0.03% (0 - 0.05)	0.03% (0 - 0.05)	0.1% (0.05 - 0.1)	0.05% (0.04 - 0.1)	0.05% (0.04 - 0.1)
Namibia	2.9% (1.5 - 5.7)	2.2% (1.2 - 3.8)	0*	3.9% (1.7 - 10.5)	0.3% (0 - 0.5)	0.2% (0 - 0.3)	0.2% (0 - 0.3)	0.03% (0.02 - 0.03)	0.2% (0.2 - 0.2)	0.2% (0.2 - 0.2)
Nigeria	0.7% (0.4 - 1.3)	1.9% (1.1 - 3.1)	1.3% (0.4 - 2.5)	0.6% (0.3 - 1.7)	0.004% (0 - 0.007)	0.003% (0 - 0.004)	0.003% (0 - 0.004)	0.006% (0.005 - 0.006)	0.007% (0.006 - 0.008)	0.007% (0.006 - 0.008)
Rwanda	13.4% (6.1 - 37.3)	7.2% (3.8 - 14.2)	0*	0.1% (0.05 - 0.3)	0.02% (0 - 0.04)	0.01% (0 - 0.02)	0.01% (0 - 0.02)	0.002% (0.001 - 0.002)	0.002% (0.001 - 0.002)	0.002% (0.001 - 0.002)
Senegal	0.03% (0.02 - 0.1)	0.4% (0.2 - 0.7)	0*	0*	0*	0*	0*	0*	0*	0*
Sierra Leone	5.7% (2.6 - 14.6)	1.4% (0.7 - 2.7)	0*	0*	0*	0*	0*	0*	0*	0*
South Africa	1.3% (0.7 - 2.4)	3.9% (2.2 - 6.4)	0.04% (0.01 - 0.1)	2.0% (0.9 - 5.6)	0.3% (0 - 0.6)	0.1% (0 - 0.2)	0.1% (0 - 0.2)	0.1% (0.1 - 0.1)	0.1% (0.1 - 0.1)	0.1% (0.1 - 0.1)
South Sudan	0.03% (0.02 - 0.1)	0*	0*	0*	0.0004% (0 - 0.0007)	0.0003% (0 - 0.0005)	0.0003% (0 - 0.0005)	0.0007% (0.0006 - 0.0008)	0.001% (0.001 - 0.001)	0.001% (0.001 - 0.001)

Country	FSW	MSM	PWID	TGW	Females 15-24	Females 25-34	Females 35-49	Males 15-24	Males 25-34	Males 35-49
Tanzania	13.3% (7.0 - 26.2)	3.5% (2.0 - 5.9)	1.8% (0.6 - 3.5)	0*	0.1% (0 - 0.1)	0.03% (0 - 0.1)	0.03% (0 - 0.1)	0.04% (0.04 - 0.05)	0.1% (0.1 - 0.1)	0.1% (0.1 - 0.1)
Togo	0.4% (0.2 - 0.7)	0.3% (0.2 - 0.6)	0*	0*	0.0008% (0 - 0.001)	0.001% (0 - 0.002)	0.001% (0 - 0.002)	0.003% (0.002 - 0.003)	0.004% (0.004 - 0.005)	0.005% (0.004 - 0.005)
Uganda	1.3% (0.7 - 2.4)	7.6% (4.2 - 13.8)	7.7% (2.4 - 17.2)	4.3% (2.0 - 11.5)	0.2% (0 - 0.4)	0.1% (0 - 0.2)	0.1% (0 - 0.2)	0.2% (0.2 - 0.2)	0.2% (0.2 - 0.2)	0.2% (0.2 - 0.2)
Zambia	11.1% (5.9 - 21.5)	18.1% (9.7 - 33.2)	7.8% (2.5 - 17.1)	16.3% (7.1 - 49.6)	0.2% (0 - 0.3)	0.1% (0 - 0.1)	0.1% (0 - 0.1)	0.1% (0.1 - 0.1)	0.2% (0.1 - 0.2)	0.2% (0.1 - 0.2)
Zimbabwe	10.2% (5.0 - 23.4)	9.1% (4.9 - 17.1)	4.4% (1.4 - 10.7)	9.3% (4.3 - 25.0)	0.2% (0 - 0.3)	0.1% (0 - 0.2)	0.1% (0 - 0.2)	0.04% (0.04 - 0.05)	0.1% (0.1 - 0.1)	0.1% (0.1 - 0.1)

Supplementary Table 8: Baseline results - Absolute impact of removing PEPFAR funded PrEP for 1-year (Primary Infections)

Cells present median projection with 95% uncertainty interval in parentheses, given to 1 significant figure after the decimal point. * No PEPFAR funded PrEP.

Country	Overall	FSW	MSM	PWID	TGW	Females 15-24	Females 25-34	Females 35-49	Males 15-24	Males 25-34	Males 35-49
Benin	4.4 (3.0 - 5.7)	0.9 (0.5 - 1.6)	3.2 (2.0 - 4.4)	0*	0*	0.008 (0 - 0.02)	0.1 (0 - 0.1)	0.02 (0 - 0.1)	0.006 (0.001 - 0.01)	0.1 (0.02 - 0.1)	0.04 (0.009 - 0.1)
Botswana	22.7 (14.2 - 35.7)	3.7 (2.4 - 5.4)	13.2 (6.2 - 25.2)	0.1 (0.03 - 0.2)	0.8 (0.3 - 1.9)	0.2 (0 - 0.8)	1.1 (0 - 2.8)	0.8 (0 - 2.2)	0.1 (0.006 - 0.4)	0.8 (0.03 - 2.4)	1.1 (0.2 - 2.3)
Burkina Faso	2.7 (1.7 - 3.9)	1.2 (0.6 - 2.0)	1.4 (0.7 - 2.4)	0*	0*	0.005 (0 - 0.01)	0.008 (0 - 0.02)	0.003 (0 - 0.009)	0.003 (0.0006 - 0.005)	0.009 (0.004 - 0.02)	0.007 (0.002 - 0.01)
Burundi	6.1 (2.7 - 12.7)	0.9 (0.5 - 1.5)	4.4 (1.4 - 10.2)	0.05 (0.02 - 0.1)	0.5 (0.1 - 1.4)	0.03 (0 - 0.1)	0.1 (0 - 0.2)	0.04 (0 - 0.1)	0.007 (0.002 - 0.01)	0.05 (0.02 - 0.1)	0.04 (0.009 - 0.1)
Cameroon	106.2 (41.0 - 232.3)	11.0 (5.8 - 19.0)	94.8 (30.1 - 220.6)	0*	0*	0.01 (0 - 0.03)	0.008 (0 - 0.02)	0.007 (0 - 0.02)	0.002 (0.0004 - 0.004)	0.002 (0.0005 - 0.005)	0.0009 (0.0002 - 0.002)
Cote d'Ivoire	39.2 (25.0 - 55.0)	3.0 (1.4 - 5.5)	33.9 (19.9 - 49.4)	0*	0*	0.3 (0 - 1.0)	0.1 (0 - 0.3)	0.2 (0 - 0.7)	0.2 (0.1 - 0.5)	0.5 (0.02 - 1.6)	0.6 (0.03 - 1.8)
Democratic Republic of the Congo	31.2 (14.2 - 63.8)	5.2 (2.7 - 9.0)	23.8 (7.6 - 55.6)	0.05 (0.02 - 0.1)	0.5 (0.1 - 1.4)	0.1 (0 - 0.3)	0.3 (0 - 0.7)	0.1 (0 - 0.4)	0.1 (0.01 - 0.1)	0.4 (0.2 - 0.7)	0.3 (0.1 - 0.6)
Eswatini	32.6 (11.7 - 55.9)	3.0 (1.9 - 4.4)	4.3 (2.0 - 8.3)	0.3 (0.1 - 0.6)	0.3 (0.1 - 0.7)	13.0 (0 - 30.8)	5.7 (0 - 13.6)	1.2 (0 - 3.2)	0.6 (0.1 - 1.2)	1.7 (0.4 - 3.6)	1.1 (0.2 - 2.3)
Ethiopia	200.3 (127.0 - 287.3)	199.7 (126.4 - 286.7)	0*	0*	0*	0.03 (0 - 0.2)	0.03 (0 - 0.1)	0.04 (0 - 0.1)	0.03 (0.006 - 0.1)	0.2 (0.008 - 0.5)	0.2 (0.009 - 0.6)
Ghana	0.2 (0.1 - 0.3)	0.02 (0.01 - 0.04)	0.1 (0.03 - 0.2)	0*	0*	0.01 (0 - 0.02)	0.01 (0 - 0.03)	0.007 (0 - 0.02)	0.005 (0.001 - 0.008)	0.02 (0.007 - 0.03)	0.01 (0.003 - 0.03)
Kenya	302.2 (215.6 - 400.0)	94.3 (50.9 - 161.3)	134.9 (78.7 - 200.9)	2.0 (0.7 - 3.2)	21.2 (7.8 - 44.7)	1.4 (0 - 6.6)	8.7 (0 - 25.0)	6.8 (0 - 19.1)	4.7 (1.0 - 9.1)	9.5 (2.1 - 19.1)	10.5 (2.3 - 22.0)
Lesotho	67.3 (42.7 - 101.4)	11.1 (7.0 - 15.9)	27.4 (12.8 - 51.9)	0.03 (0.01 - 0.1)	7.2 (2.9 - 16.4)	1.8 (0 - 5.1)	6.0 (0 - 14.0)	3.1 (0 - 8.6)	1.3 (0.3 - 2.5)	4.7 (1.0 - 8.9)	2.5 (0.1 - 7.4)
Liberia	164.9 (62.5 - 363.9)	8.8 (4.6 - 15.1)	132.3 (41.9 - 308.3)	5.7 (1.9 - 9.2)	16.4 (4.7 - 45.2)	0.2 (0 - 0.5)	0.1 (0 - 0.3)	0.1 (0 - 0.2)	0.1 (0.03 - 0.3)	0.2 (0.1 - 0.3)	0.1 (0.03 - 0.3)
Malawi	191.0 (91.6 - 386.4)	61.7 (39.1 - 88.2)	84.1 (17.1 - 220.1)	0.6 (0.2 - 1.0)	32.5 (6.2 - 99.2)	2.9 (0 - 7.0)	1.1 (0 - 3.1)	0.9 (0 - 2.5)	0.6 (0.1 - 1.3)	2.8 (0.6 - 5.2)	1.0 (0.04 - 2.9)
Mali	54.1 (34.1 - 73.9)	3.9 (2.1 - 6.7)	50.1 (30.2 - 69.7)	0*	0*	0*	0*	0*	0*	0*	0*
Mozambique	139.6 (93.0 - 200.7)	35.3 (22.4 - 50.6)	56.6 (26.3 - 107.2)	3.0 (1.0 - 4.9)	3.6 (1.4 - 8.0)	10.3 (0 - 25.3)	5.9 (0 - 16.1)	6.5 (0 - 18.5)	5.8 (1.3 - 11.4)	4.0 (0.8 - 8.7)	4.3 (0.9 - 9.1)
Namibia	39.7 (24.1 - 57.5)	9.4 (6.0 - 13.5)	6.8 (3.2 - 12.9)	0*	4.5 (1.8 - 10.2)	6.3 (0 - 14.6)	1.8 (0 - 4.9)	3.5 (0 - 10.2)	0.04 (0.007 - 0.1)	1.4 (0.1 - 4.1)	4.3 (1.0 - 8.0)
Nigeria	946.5 (646.5 - 1192.2)	87.0 (46.2 - 150.3)	700.7 (432.3 - 922.9)	81.1 (27.6 - 131.6)	66.5 (37.1 - 103.4)	0.5 (0 - 1.2)	1.5 (0 - 3.3)	0.7 (0 - 1.6)	0.5 (0.1 - 0.9)	1.9 (0.6 - 3.4)	1.2 (0.3 - 2.6)
Rwanda	256.8 (173.1 - 361.1)	157.1 (99.4 - 225.6)	94.7 (44.2 - 179.6)	0*	0.5 (0.2 - 1.2)	0.2 (0 - 0.7)	0.1 (0 - 0.4)	0.2 (0 - 0.7)	0.02 (0.003 - 0.03)	0.1 (0.01 - 0.1)	0.03 (0.001 - 0.1)
Senegal	2.6 (1.4 - 4.3)	0.2 (0.1 - 0.4)	2.4 (1.2 - 4.1)	0*	0*	0*	0*	0*	0*	0*	0*
Sierra Leone	52.0 (27.6 - 91.6)	22.7 (12.0 - 39.0)	28.1 (9.0 - 65.6)	0*	0*	0*	0*	0*	0*	0*	0*
South Africa	932.0 (553.6 - 1284.4)	49.3 (21.2 - 90.5)	321.7 (184.3 - 491.4)	4.7 (1.6 - 6.8)	133.4 (34.5 - 304.5)	234.4 (0 - 411.9)	35.9 (0 - 63.0)	45.1 (0 - 79.2)	16.7 (10.7 - 23.3)	36.3 (30.2 - 42.0)	45.5 (37.9 - 52.6)
South Sudan	0.6 (0.4 - 0.8)	0.5 (0.3 - 0.6)	0*	0*	0*	0.009 (0 - 0.02)	0.02 (0 - 0.03)	0.008 (0 - 0.02)	0.01 (0.003 - 0.02)	0.03 (0.02 - 0.05)	0.1 (0.03 - 0.1)

Country	Overall	FSW	MSM	PWID	TGW	Females 15-24	Females 25-34	Females 35-49	Males 15-24	Males 25-34	Males 35-49
Tanzania	1165.4 (693.0 - 1884.8)	877.1 (439.2 - 1584.2)	202.3 (94.7 - 383.4)	21.1 (7.2 - 34.4)	0*	10.9 (0 - 25.8)	7.4 (0 - 18.6)	4.6 (0 - 12.8)	2.2 (0.1 - 6.4)	12.2 (2.7 - 23.2)	9.9 (2.2 - 19.2)
Togo	6.2 (3.9 - 9.3)	1.4 (0.7 - 2.4)	4.6 (2.5 - 7.6)	0*	0*	0.007 (0 - 0.02)	0.02 (0 - 0.04)	0.01 (0 - 0.03)	0.009 (0.002 - 0.02)	0.05 (0.02 - 0.1)	0.04 (0.009 - 0.1)
Uganda	678.8 (431.6 - 1029.9)	40.0 (25.0 - 58.6)	303.4 (141.5 - 574.3)	53.7 (18.2 - 87.4)	62.5 (25.3 - 141.5)	73.2 (0 - 155.4)	21.8 (0 - 56.8)	9.3 (0 - 26.6)	6.6 (0.3 - 19.1)	61.4 (18.7 - 106.3)	34.3 (7.7 - 63.1)
Zambia	721.2 (469.1 - 1111.0)	160.0 (101.1 - 229.5)	313.6 (146.5 - 594.8)	25.8 (8.8 - 41.7)	98.5 (40.0 - 223.6)	33.2 (0 - 70.0)	19.5 (0 - 43.4)	18.9 (0 - 44.3)	2.5 (0.5 - 5.3)	11.1 (2.4 - 22.0)	28.8 (8.0 - 50.5)
Zimbabwe	368.6 (248.1 - 539.7)	131.4 (79.1 - 201.4)	119.6 (55.9 - 226.4)	11.2 (3.8 - 18.2)	47.0 (19.1 - 106.2)	18.3 (0 - 41.7)	9.1 (0 - 23.6)	9.7 (0 - 26.4)	0.8 (0.2 - 1.8)	5.0 (1.1 - 9.9)	7.9 (1.7 - 15.4)

Supplementary Table 9: Baseline results - Absolute impact of removing PEPFAR funded PrEP for 1-year (Primary Infections over 1 year and Secondary Infections over 5 years)

Cells present median projection with 95% uncertainty interval in parentheses, given to 1 significant figure after the decimal point. * No PEPFAR funded PrEP.

Country	Overall	FSW	MSM	PWID	TGW	Females 15-24	Females 25-34	Females 35-49	Males 15-24	Males 25-34	Males 35-49
Benin	6.9 (4.7 - 9.5)	1.6 (0.8 - 2.8)	5.0 (3.0 - 7.4)	0*	0*	0.01 (0 - 0.02)	0.1 (0 - 0.1)	0.03 (0 - 0.1)	0.007 (0.002 - 0.01)	0.1 (0.03 - 0.1)	0.1 (0.01 - 0.1)
Botswana	30.3 (19.0 - 48.3)	6.5 (3.8 - 10.4)	16.7 (7.6 - 32.8)	0.1 (0.05 - 0.3)	1.3 (0.5 - 3.2)	0.3 (0 - 1.2)	1.2 (0 - 3.1)	0.9 (0 - 2.4)	0.2 (0.007 - 0.5)	1.0 (0.04 - 2.9)	1.3 (0.3 - 2.7)
Burkina Faso	4.4 (2.7 - 6.5)	2.0 (1.0 - 3.5)	2.2 (1.1 - 3.9)	0*	0*	0.006 (0 - 0.01)	0.009 (0 - 0.02)	0.004 (0 - 0.01)	0.003 (0.0007 - 0.006)	0.01 (0.005 - 0.02)	0.009 (0.002 - 0.02)
Burundi	9.6 (4.3 - 17.9)	1.5 (0.8 - 2.6)	6.8 (2.1 - 14.3)	0.1 (0.04 - 0.2)	0.9 (0.3 - 1.8)	0.04 (0 - 0.1)	0.1 (0 - 0.2)	0.04 (0 - 0.1)	0.008 (0.002 - 0.02)	0.1 (0.03 - 0.1)	0.1 (0.01 - 0.1)
Cameroon	166.6 (64.5 - 329.7)	18.6 (9.7 - 32.7)	147.1 (46.0 - 309.7)	0*	0*	0.02 (0 - 0.04)	0.009 (0 - 0.03)	0.008 (0 - 0.02)	0.002 (0.0005 - 0.004)	0.003 (0.0007 - 0.006)	0.001 (0.0002 - 0.003)
Cote d'Ivoire	60.7 (37.8 - 90.3)	5.0 (2.4 - 9.5)	52.6 (30.1 - 81.8)	0*	0*	0.4 (0 - 1.2)	0.1 (0 - 0.3)	0.2 (0 - 0.8)	0.3 (0.1 - 0.6)	0.7 (0.03 - 2.0)	0.8 (0.03 - 2.4)
Democratic Republic of the Congo	49.2 (22.4 - 91.0)	8.8 (4.6 - 15.5)	37.0 (11.6 - 77.8)	0.3 (0.1 - 0.7)	0.9 (0.3 - 1.8)	0.2 (0 - 0.3)	0.4 (0 - 0.8)	0.2 (0 - 0.4)	0.1 (0.02 - 0.1)	0.6 (0.2 - 1.0)	0.4 (0.1 - 0.8)
Eswatini	43.5 (16.1 - 77.0)	5.2 (3.1 - 8.5)	5.5 (2.5 - 10.8)	0.5 (0.2 - 0.9)	0.5 (0.2 - 1.2)	18.6 (0 - 46.6)	6.2 (0 - 14.9)	1.3 (0 - 3.5)	0.7 (0.2 - 1.5)	2.0 (0.4 - 4.2)	1.3 (0.3 - 2.7)
Ethiopia	345.9 (202.0 - 556.6)	345.2 (201.2 - 555.9)	0*	0*	0*	0.05 (0 - 0.2)	0.03 (0 - 0.1)	0.04 (0 - 0.1)	0.04 (0.007 - 0.1)	0.2 (0.009 - 0.6)	0.2 (0.01 - 0.7)
Ghana	0.3 (0.2 - 0.5)	0.04 (0.02 - 0.1)	0.2 (0.1 - 0.3)	0*	0*	0.01 (0 - 0.03)	0.02 (0 - 0.04)	0.008 (0 - 0.02)	0.005 (0.001 - 0.01)	0.02 (0.009 - 0.04)	0.02 (0.004 - 0.04)
Kenya	426.4 (295.9 - 598.4)	163.4 (82.0 - 304.8)	169.6 (96.3 - 266.3)	3.9 (1.3 - 8.5)	30.7 (11.7 - 56.1)	2.1 (0 - 9.7)	9.5 (0 - 27.3)	7.4 (0 - 20.9)	5.7 (1.3 - 11.3)	11.1 (2.4 - 22.4)	12.3 (2.7 - 25.8)
Lesotho	91.2 (57.9 - 139.5)	19.2 (11.2 - 31.0)	34.5 (15.7 - 67.7)	0.05 (0.02 - 0.1)	11.5 (4.1 - 28.1)	2.6 (0 - 7.6)	6.6 (0 - 15.3)	3.4 (0 - 9.4)	1.6 (0.3 - 3.1)	5.5 (1.2 - 10.5)	3.0 (0.1 - 8.7)
Liberia	273.9 (113.1 - 523.5)	14.9 (7.8 - 26.1)	205.2 (64.3 - 431.0)	20.4 (6.3 - 54.5)	28.7 (9.5 - 58.4)	0.3 (0 - 0.6)	0.2 (0 - 0.3)	0.1 (0 - 0.2)	0.2 (0.04 - 0.3)	0.2 (0.1 - 0.4)	0.2 (0.04 - 0.4)
Malawi	285.1 (139.4 - 577.7)	106.6 (62.1 - 171.8)	106.1 (21.5 - 284.8)	0.9 (0.3 - 2.1)	51.9 (9.3 - 185.0)	4.1 (0 - 10.6)	1.2 (0 - 3.4)	0.9 (0 - 2.7)	0.8 (0.2 - 1.7)	3.3 (0.7 - 6.1)	1.1 (0.05 - 3.4)
Mali	84.4 (52.1 - 123.7)	6.6 (3.4 - 11.5)	77.6 (45.6 - 116.7)	0*	0*	0*	0*	0*	0*	0*	0*
Mozambique	194.1 (129.1 - 280.2)	61.0 (35.5 - 98.4)	71.3 (32.4 - 140.1)	3.9 (1.3 - 7.0)	5.7 (2.0 - 13.8)	14.7 (0 - 38.2)	6.4 (0 - 17.6)	7.1 (0 - 20.2)	7.0 (1.5 - 14.1)	4.7 (1.0 - 10.3)	5.1 (1.1 - 10.7)
Namibia	56.3 (34.5 - 81.9)	16.3 (9.5 - 26.3)	8.6 (3.9 - 16.8)	0*	7.2 (2.5 - 17.5)	9.0 (0 - 22.3)	1.9 (0 - 5.4)	3.8 (0 - 11.2)	0.04 (0.008 - 0.1)	1.7 (0.1 - 4.8)	5.0 (1.1 - 9.3)
Nigeria	1810.3 (1205.2 - 2697.8)	147.8 (77.6 - 259.2)	1082.8 (652.4 - 1563.7)	394.6 (117.8 - 1167.7)	134.8 (74.1 - 224.2)	0.6 (0 - 1.6)	1.7 (0 - 3.8)	0.7 (0 - 1.8)	0.6 (0.1 - 1.1)	2.5 (0.8 - 4.4)	1.6 (0.4 - 3.4)
Rwanda	400.3 (258.2 - 593.2)	271.2 (158.3 - 436.8)	119.2 (54.2 - 234.7)	0*	0.8 (0.3 - 2.0)	0.3 (0 - 1.1)	0.1 (0 - 0.4)	0.2 (0 - 0.8)	0.02 (0.004 - 0.04)	0.1 (0.02 - 0.1)	0.03 (0.001 - 0.1)
Senegal	4.0 (2.1 - 7.0)	0.3 (0.1 - 0.7)	3.7 (1.8 - 6.7)	0*	0*	0*	0*	0*	0*	0*	0*
Sierra Leone	84.2 (44.8 - 138.6)	38.5 (20.1 - 67.5)	43.5 (13.7 - 91.7)	0*	0*	0*	0*	0*	0*	0*	0*
South Africa	1226.4 (722.3 - 1720.5)	85.2 (35.1 - 169.4)	405.0 (225.8 - 648.0)	16.6 (5.7 - 26.3)	168.5 (53.2 - 333.1)	332.8 (0 - 652.5)	39.1 (0 - 68.9)	49.2 (0 - 86.6)	20.2 (12.6 - 29.3)	42.5 (35.0 - 49.9)	53.2 (43.9 - 62.5)
South Sudan	0.9 (0.6 - 1.4)	0.8 (0.5 - 1.3)	0*	0*	0*	0.01 (0 - 0.03)	0.02 (0 - 0.04)	0.009 (0 - 0.02)	0.01 (0.004 - 0.02)	0.04 (0.02 - 0.1)	0.1 (0.03 - 0.1)
Tanzania	1901.7 (1060.6 - 3368.4)	1520.8 (714.1 - 2971.0)	255.4 (116.4 - 501.7)	41.4 (13.4 - 101.6)	0*	15.6 (0 - 39.2)	8.0 (0 - 20.3)	5.0 (0 - 13.9)	2.7 (0.1 - 7.9)	14.3 (3.2 - 27.3)	11.6 (2.6 - 22.6)

Togo	9.8 (6.1 - 15.2)	2.3 (1.2 - 4.1)	7.2 (3.8 - 12.5)	0*	0*	0.009 (0 - 0.02)	0.02 (0 - 0.05)	0.01 (0 - 0.03)	0.01 (0.002 - 0.02)	0.1 (0.02 - 0.1)	0.1 (0.01 - 0.1)
Uganda	911.6 (571.8 - 1407.9)	69.2 (39.7 - 113.4)	383.3 (174.3 - 751.2)	75.9 (25.4 - 144.3)	100.2 (35.4 - 243.5)	104.3 (0 - 237.7)	23.8 (0 - 62.0)	10.2 (0 - 29.1)	8.0 (0.3 - 23.5)	71.9 (21.8 - 124.9)	40.1 (9.0 - 74.2)
Zambia	1034.2 (662.8 - 1604.4)	276.6 (160.4 - 446.5)	396.2 (180.5 - 772.9)	39.1 (13.1 - 74.4)	157.8 (56.2 - 382.2)	47.3 (0 - 107.1)	21.3 (0 - 47.5)	20.6 (0 - 48.4)	3.0 (0.6 - 6.6)	13.0 (2.8 - 25.8)	33.7 (9.4 - 59.3)
Zimbabwe	551.7 (359.5 - 820.5)	227.1 (126.6 - 386.1)	151.1 (68.7 - 295.4)	16.8 (5.6 - 34.0)	75.2 (26.8 - 181.9)	26.2 (0 - 63.4)	9.9 (0 - 25.7)	10.6 (0 - 28.8)	1.0 (0.2 - 2.2)	5.8 (1.3 - 11.6)	9.2 (2.0 - 18.1)

Supplementary Table 10: Sensitivity Analysis – Absolute impact of removing PEPFAR funded PrEP for 1-year (Primary Infections)

Cells present median projection with 95% uncertainty interval in parentheses, given to 1 significant figure after the decimal point. * No PEPFAR funded PrEP.

Country	Overall	FSW	MSM	PWID	TGW	Females 15-24	Females 25-34	Females 35-49	Males 15-24	Males 25-34	Males 35-49
Benin	8.7 (5.7 - 11.4)	0.9 (0.5 - 1.6)	7.6 (4.6 - 10.2)	0*	0*	0.008 (0 - 0.02)	0.1 (0 - 0.1)	0.02 (0 - 0.1)	0.003 (0.0007 - 0.005)	0.03 (0.01 - 0.05)	0.02 (0.004 - 0.04)
Botswana	49.1 (26.5 - 86.9)	3.7 (2.4 - 5.4)	40.9 (19.1 - 77.8)	0.1 (0.03 - 0.2)	0.8 (0.3 - 1.9)	0.2 (0 - 0.8)	1.1 (0 - 2.8)	0.8 (0 - 2.2)	0.1 (0.003 - 0.2)	0.4 (0.02 - 1.2)	0.5 (0.1 - 1.2)
Burkina Faso	3.8 (2.4 - 5.7)	1.2 (0.6 - 2.0)	2.6 (1.3 - 4.3)	0*	0*	0.005 (0 - 0.01)	0.008 (0 - 0.02)	0.003 (0 - 0.009)	0.001 (0.0003 - 0.002)	0.005 (0.002 - 0.008)	0.004 (0.0008 - 0.007)
Burundi	16.0 (5.9 - 36.0)	0.9 (0.5 - 1.5)	14.4 (4.6 - 33.6)	0.05 (0.02 - 0.1)	0.5 (0.1 - 1.4)	0.03 (0 - 0.1)	0.1 (0 - 0.2)	0.04 (0 - 0.1)	0.004 (0.0008 - 0.006)	0.02 (0.01 - 0.04)	0.02 (0.005 - 0.04)
Cameroon	106.6 (41.2 - 233.1)	11.0 (5.8 - 19.0)	95.2 (30.2 - 221.4)	0*	0*	0.01 (0 - 0.03)	0.008 (0 - 0.02)	0.007 (0 - 0.02)	0.0009 (0.0002 - 0.002)	0.001 (0.0003 - 0.002)	0.0004 (9e-05 - 0.001)
Cote d'Ivoire	129.1 (77.7 - 186.4)	3.0 (1.4 - 5.5)	124.5 (73.2 - 181.7)	0*	0*	0.3 (0 - 1.0)	0.1 (0 - 0.3)	0.2 (0 - 0.7)	0.1 (0.03 - 0.2)	0.3 (0.01 - 0.8)	0.3 (0.01 - 0.9)
Democratic Republic of the Congo	110.3 (39.4 - 249.2)	5.2 (2.7 - 9.0)	103.4 (32.9 - 241.5)	0.05 (0.02 - 0.1)	0.5 (0.1 - 1.4)	0.1 (0 - 0.3)	0.3 (0 - 0.7)	0.1 (0 - 0.4)	0.03 (0.007 - 0.1)	0.2 (0.1 - 0.4)	0.1 (0.03 - 0.3)
Eswatini	63.9 (33.1 - 103.0)	3.0 (1.9 - 4.4)	36.7 (17.1 - 69.8)	0.3 (0.1 - 0.6)	0.3 (0.1 - 0.7)	13.0 (0 - 30.8)	5.7 (0 - 13.6)	1.2 (0 - 3.2)	0.3 (0.1 - 0.6)	0.9 (0.2 - 1.8)	0.5 (0.1 - 1.2)
Ethiopia	216.4 (142.1 - 303.7)	199.7 (126.4 - 286.7)	15.5 (7.2 - 29.3)	0*	0*	0.03 (0 - 0.2)	0.03 (0 - 0.1)	0.04 (0 - 0.1)	0.006 (0.001 - 0.01)	0.04 (0.002 - 0.1)	0.04 (0.002 - 0.1)
Ghana	4.1 (1.4 - 9.4)	0.02 (0.01 - 0.04)	4.0 (1.3 - 9.3)	0*	0*	0.01 (0 - 0.02)	0.01 (0 - 0.03)	0.007 (0 - 0.02)	0.002 (0.0005 - 0.004)	0.009 (0.003 - 0.01)	0.007 (0.002 - 0.01)
Kenya	554.5 (370.1 - 762.7)	94.3 (50.9 - 161.3)	399.3 (232.8 - 594.8)	2.0 (0.7 - 3.2)	21.2 (7.8 - 44.7)	1.4 (0 - 6.6)	8.7 (0 - 25.0)	6.8 (0 - 19.1)	2.4 (0.5 - 4.5)	4.8 (1.0 - 9.5)	5.3 (1.1 - 11.0)
Lesotho	126.8 (71.8 - 216.5)	11.1 (7.0 - 15.9)	91.8 (43.0 - 174.1)	0.03 (0.01 - 0.1)	7.2 (2.9 - 16.4)	1.8 (0 - 5.1)	6.0 (0 - 14.0)	3.1 (0 - 8.6)	0.7 (0.1 - 1.3)	2.4 (0.5 - 4.5)	1.3 (0.1 - 3.7)
Liberia	221.1 (80.2 - 494.7)	8.8 (4.6 - 15.1)	188.7 (59.8 - 439.9)	5.7 (1.9 - 9.2)	16.4 (4.7 - 45.2)	0.2 (0 - 0.5)	0.1 (0 - 0.3)	0.1 (0 - 0.2)	0.1 (0.02 - 0.1)	0.1 (0.03 - 0.1)	0.1 (0.01 - 0.1)
Malawi	224.6 (98.1 - 476.8)	61.7 (39.1 - 88.2)	120.0 (24.5 - 314.1)	0.6 (0.2 - 1.0)	32.5 (6.2 - 99.2)	2.9 (0 - 7.0)	1.1 (0 - 3.1)	0.9 (0 - 2.5)	0.3 (0.1 - 0.7)	1.4 (0.3 - 2.6)	0.5 (0.02 - 1.4)
Mali	54.1 (34.1 - 73.9)	3.9 (2.1 - 6.7)	50.1 (30.2 - 69.7)	0*	0*	0*	0*	0*	0*	0*	0*
Mozambique	303.9 (174.8 - 514.5)	35.3 (22.4 - 50.6)	229.1 (106.4 - 434.0)	3.0 (1.0 - 4.9)	3.6 (1.4 - 8.0)	10.3 (0 - 25.3)	5.9 (0 - 16.1)	6.5 (0 - 18.5)	2.9 (0.6 - 5.7)	2.0 (0.4 - 4.4)	2.2 (0.5 - 4.5)
Namibia	88.6 (51.2 - 146.7)	9.4 (6.0 - 13.5)	58.6 (27.3 - 111.2)	0*	4.5 (1.8 - 10.2)	6.3 (0 - 14.6)	1.8 (0 - 4.9)	3.5 (0 - 10.2)	0.02 (0.003 - 0.04)	0.7 (0.03 - 2.1)	2.1 (0.5 - 4.0)
Nigeria	1247.0 (832.8 - 1583.4)	87.0 (46.2 - 150.3)	1002.4 (618.5 - 1320.2)	81.1 (27.6 - 131.6)	66.5 (37.1 - 103.4)	0.5 (0 - 1.2)	1.5 (0 - 3.3)	0.7 (0 - 1.6)	0.2 (0.1 - 0.5)	1.0 (0.3 - 1.7)	0.6 (0.1 - 1.3)
Rwanda	259.4 (174.6 - 365.4)	157.1 (99.4 - 225.6)	97.2 (45.4 - 184.4)	0*	0.5 (0.2 - 1.2)	0.2 (0 - 0.7)	0.1 (0 - 0.4)	0.2 (0 - 0.7)	0.008 (0.002 - 0.02)	0.03 (0.007 - 0.1)	0.01 (0.0006 - 0.04)
Senegal	2.6 (1.4 - 4.3)	0.2 (0.1 - 0.4)	2.4 (1.2 - 4.1)	0*	0*	0*	0*	0*	0*	0*	0*
Sierra Leone	52.0 (27.6 - 91.6)	22.7 (12.0 - 39.0)	28.1 (9.0 - 65.6)	0*	0*	0*	0*	0*	0*	0*	0*
South Africa	1837.1 (1164.9 - 2585.8)	49.3 (21.2 - 90.5)	1277.8 (732.0 - 1951.6)	4.7 (1.6 - 6.8)	133.4 (34.5 - 304.5)	234.4 (0 - 411.9)	35.9 (0 - 63.0)	45.1 (0 - 79.2)	8.3 (5.3 - 11.6)	18.2 (15.1 - 21.0)	22.8 (18.9 - 26.3)
South Sudan	2.9 (1.6 - 5.1)	0.5 (0.3 - 0.6)	2.4 (1.1 - 4.6)	0*	0*	0.009 (0 - 0.02)	0.02 (0 - 0.03)	0.008 (0 - 0.02)	0.002 (0.0006 - 0.004)	0.006 (0.003 - 0.01)	0.01 (0.006 - 0.02)
Tanzania	1599.2 (975.6 - 2446.0)	877.1 (439.2 - 1584.2)	619.7 (290.3 - 1174.7)	21.1 (7.2 - 34.4)	0*	10.9 (0 - 25.8)	7.4 (0 - 18.6)	4.6 (0 - 12.8)	1.1 (0.05 - 3.2)	6.1 (1.4 - 11.6)	5.0 (1.1 - 9.6)

Country	Overall	FSW	MSM	PWID	TGW	Females 15-24	Females 25-34	Females 35-49	Males 15-24	Males 25-34	Males 35-49
Togo	13.9 (8.1 - 21.7)	1.4 (0.7 - 2.4)	12.4 (6.7 - 20.2)	0*	0*	0.007 (0 - 0.02)	0.02 (0 - 0.04)	0.01 (0 - 0.03)	0.005 (0.001 - 0.009)	0.02 (0.009 - 0.04)	0.02 (0.004 - 0.04)
Uganda	1669.1 (902.1 - 2938.9)	40.0 (25.0 - 58.6)	1349.9 (629.6 - 2555.0)	53.7 (18.2 - 87.4)	62.5 (25.3 - 141.5)	73.2 (0 - 155.4)	21.8 (0 - 56.8)	9.3 (0 - 26.6)	3.3 (0.1 - 9.5)	30.7 (9.3 - 53.2)	17.1 (3.9 - 31.6)
Zambia	1027.2 (613.1 - 1703.8)	160.0 (101.1 - 229.5)	642.1 (299.9 - 1218.0)	25.8 (8.8 - 41.7)	98.5 (40.0 - 223.6)	33.2 (0 - 70.0)	19.5 (0 - 43.4)	18.9 (0 - 44.3)	1.2 (0.3 - 2.7)	5.6 (1.2 - 11.0)	14.4 (4.0 - 25.2)
Zimbabwe	485.1 (308.8 - 756.7)	131.4 (79.1 - 201.4)	243.4 (113.7 - 460.7)	11.2 (3.8 - 18.2)	47.0 (19.1 - 106.2)	18.3 (0 - 41.7)	9.1 (0 - 23.6)	9.7 (0 - 26.4)	0.4 (0.1 - 0.9)	2.5 (0.5 - 4.9)	3.9 (0.9 - 7.7)

Supplementary Table 11: Sensitivity Analysis – Absolute impact of removing PEPFAR funded PrEP for 1-year (Primary Infections over 1 year and Secondary Infections over 5 years).

Cells present median projection with 95% uncertainty interval in parentheses, given to 1 significant figure after the decimal point. * No PEPFAR funded PrEP.

Country	Overall	FSW	MSM	PWID	TGW	Females 15-24	Females 25-34	Females 35-49	Males 15-24	Males 25-34	Males 35-49
Benin	13.5 (8.7 - 19.1)	1.6 (0.8 - 2.8)	11.7 (7.0 - 17.2)	0*	0*	0.01 (0 - 0.02)	0.1 (0 - 0.1)	0.03 (0 - 0.1)	0.004 (0.0008 - 0.006)	0.04 (0.01 - 0.1)	0.03 (0.006 - 0.1)
Botswana	63.8 (34.3 - 115.2)	6.5 (3.8 - 10.4)	51.6 (23.4 - 101.4)	0.1 (0.05 - 0.3)	1.3 (0.5 - 3.2)	0.3 (0 - 1.2)	1.2 (0 - 3.1)	0.9 (0 - 2.4)	0.1 (0.004 - 0.3)	0.5 (0.02 - 1.4)	0.6 (0.1 - 1.4)
Burkina Faso	6.1 (3.8 - 9.5)	2.0 (1.0 - 3.5)	4.0 (2.0 - 7.1)	0*	0*	0.006 (0 - 0.01)	0.009 (0 - 0.02)	0.004 (0 - 0.01)	0.001 (0.0003 - 0.003)	0.006 (0.002 - 0.01)	0.005 (0.001 - 0.009)
Burundi	25.1 (9.2 - 50.5)	1.5 (0.8 - 2.6)	22.3 (7.0 - 47.1)	0.1 (0.04 - 0.2)	0.9 (0.3 - 1.8)	0.04 (0 - 0.1)	0.1 (0 - 0.2)	0.04 (0 - 0.1)	0.004 (0.0009 - 0.008)	0.03 (0.01 - 0.1)	0.03 (0.006 - 0.1)
Cameroon	167.2 (64.7 - 330.9)	18.6 (9.7 - 32.7)	147.6 (46.1 - 310.9)	0*	0*	0.02 (0 - 0.04)	0.009 (0 - 0.03)	0.008 (0 - 0.02)	0.001 (0.0002 - 0.002)	0.002 (0.0003 - 0.003)	0.0006 (0.0001 - 0.001)
Cote d'Ivoire	200.4 (117.8 - 307.7)	5.0 (2.4 - 9.5)	193.3 (110.8 - 300.4)	0*	0*	0.4 (0 - 1.2)	0.1 (0 - 0.3)	0.2 (0 - 0.8)	0.1 (0.03 - 0.3)	0.4 (0.02 - 1.0)	0.4 (0.02 - 1.2)
Democratic Republic of the Congo	172.1 (61.3 - 349.9)	8.8 (4.6 - 15.5)	160.5 (50.4 - 337.6)	0.3 (0.1 - 0.7)	0.9 (0.3 - 1.8)	0.2 (0 - 0.3)	0.4 (0 - 0.8)	0.2 (0 - 0.4)	0.04 (0.009 - 0.1)	0.3 (0.1 - 0.5)	0.2 (0.04 - 0.4)
Eswatini	83.5 (42.9 - 136.9)	5.2 (3.1 - 8.5)	46.2 (21.0 - 90.9)	0.5 (0.2 - 0.9)	0.5 (0.2 - 1.2)	18.6 (0 - 46.6)	6.2 (0 - 14.9)	1.3 (0 - 3.5)	0.4 (0.1 - 0.8)	1.0 (0.2 - 2.1)	0.6 (0.1 - 1.4)
Ethiopia	366.4 (221.4 - 578.2)	345.2 (201.2 - 555.9)	19.5 (8.9 - 38.2)	0*	0*	0.05 (0 - 0.2)	0.03 (0 - 0.1)	0.04 (0 - 0.1)	0.007 (0.001 - 0.02)	0.04 (0.002 - 0.1)	0.05 (0.002 - 0.1)
Ghana	6.3 (2.1 - 13.2)	0.04 (0.02 - 0.1)	6.2 (2.0 - 13.1)	0*	0*	0.01 (0 - 0.03)	0.02 (0 - 0.04)	0.008 (0 - 0.02)	0.003 (0.0006 - 0.005)	0.01 (0.004 - 0.02)	0.01 (0.002 - 0.02)
Kenya	746.6 (492.6 - 1064.8)	163.4 (82.0 - 304.8)	501.9 (285.0 - 788.4)	3.9 (1.3 - 8.5)	30.7 (11.7 - 56.1)	2.1 (0 - 9.7)	9.5 (0 - 27.3)	7.4 (0 - 20.9)	2.8 (0.6 - 5.6)	5.6 (1.2 - 11.2)	6.2 (1.3 - 12.9)
Lesotho	167.0 (94.1 - 290.0)	19.2 (11.2 - 31.0)	115.8 (52.7 - 226.9)	0.05 (0.02 - 0.1)	11.5 (4.1 - 28.1)	2.6 (0 - 7.6)	6.6 (0 - 15.3)	3.4 (0 - 9.4)	0.8 (0.2 - 1.6)	2.8 (0.6 - 5.2)	1.5 (0.1 - 4.3)
Liberia	360.9 (141.0 - 706.9)	14.9 (7.8 - 26.1)	292.8 (91.8 - 615.0)	20.4 (6.3 - 54.5)	28.7 (9.5 - 58.4)	0.3 (0 - 0.6)	0.2 (0 - 0.3)	0.1 (0 - 0.2)	0.1 (0.02 - 0.2)	0.1 (0.04 - 0.2)	0.1 (0.02 - 0.2)
Malawi	327.7 (148.8 - 692.2)	106.6 (62.1 - 171.8)	151.4 (30.7 - 406.4)	0.9 (0.3 - 2.1)	51.9 (9.3 - 185.0)	4.1 (0 - 10.6)	1.2 (0 - 3.4)	0.9 (0 - 2.7)	0.4 (0.1 - 0.8)	1.6 (0.4 - 3.1)	0.6 (0.02 - 1.7)
Mali	84.4 (52.1 - 123.7)	6.6 (3.4 - 11.5)	77.6 (45.6 - 116.7)	0*	0*	0*	0*	0*	0*	0*	0*
Mozambique	401.9 (232.9 - 688.2)	61.0 (35.5 - 98.4)	288.8 (131.1 - 567.4)	3.9 (1.3 - 7.0)	5.7 (2.0 - 13.8)	14.7 (0 - 38.2)	6.4 (0 - 17.6)	7.1 (0 - 20.2)	3.5 (0.8 - 7.1)	2.3 (0.5 - 5.1)	2.5 (0.5 - 5.3)
Namibia	118.4 (68.5 - 198.0)	16.3 (9.5 - 26.3)	73.9 (33.4 - 145.4)	0*	7.2 (2.5 - 17.5)	9.0 (0 - 22.3)	1.9 (0 - 5.4)	3.8 (0 - 11.2)	0.02 (0.004 - 0.1)	0.8 (0.04 - 2.4)	2.5 (0.6 - 4.7)
Nigeria	2281.7 (1508.9 - 3289.2)	147.8 (77.6 - 259.2)	1549.1 (933.3 - 2237.0)	394.6 (117.8 - 1167.7)	134.8 (74.1 - 224.2)	0.6 (0 - 1.6)	1.7 (0 - 3.8)	0.7 (0 - 1.8)	0.3 (0.1 - 0.5)	1.3 (0.4 - 2.2)	0.8 (0.2 - 1.7)
Rwanda	403.6 (260.1 - 598.4)	271.2 (158.3 - 436.8)	122.4 (55.6 - 241.0)	0*	0.8 (0.3 - 2.0)	0.3 (0 - 1.1)	0.1 (0 - 0.4)	0.2 (0 - 0.8)	0.009 (0.002 - 0.02)	0.04 (0.009 - 0.1)	0.02 (0.0007 - 0.05)
Senegal	4.0 (2.1 - 7.0)	0.3 (0.1 - 0.7)	3.7 (1.8 - 6.7)	0*	0*	0*	0*	0*	0*	0*	0*
Sierra Leone	84.2 (44.8 - 138.6)	38.5 (20.1 - 67.5)	43.5 (13.7 - 91.7)	0*	0*	0*	0*	0*	0*	0*	0*
South Africa	2370.4 (1479.7 - 3450.4)	85.2 (35.1 - 169.4)	1608.6 (896.7 - 2573.4)	16.6 (5.7 - 26.3)	168.5 (53.2 - 333.1)	332.8 (0 - 652.5)	39.1 (0 - 68.9)	49.2 (0 - 86.6)	10.1 (6.3 - 14.7)	21.2 (17.5 - 24.9)	26.6 (22.0 - 31.2)
South Sudan	3.9 (2.2 - 6.9)	0.8 (0.5 - 1.3)	3.1 (1.4 - 6.0)	0*	0*	0.01 (0 - 0.03)	0.02 (0 - 0.04)	0.009 (0 - 0.02)	0.003 (0.0008 - 0.005)	0.008 (0.004 - 0.01)	0.01 (0.007 - 0.02)

Country	Overall	FSW	MSM	PWID	TGW	Females 15-24	Females 25-34	Females 35-49	Males 15-24	Males 25-34	Males 35-49
Tanzania	2456.6 (1438.7 - 4023.2)	1520.8 (714.1 - 2971.0)	782.4 (356.7 - 1537.4)	41.4 (13.4 - 101.6)	0*	15.6 (0 - 39.2)	8.0 (0 - 20.3)	5.0 (0 - 13.9)	1.3 (0.1 - 3.9)	7.2 (1.6 - 13.6)	5.8 (1.3 - 11.3)
Togo	21.8 (12.4 - 35.7)	2.3 (1.2 - 4.1)	19.2 (10.0 - 33.1)	0*	0*	0.009 (0 - 0.02)	0.02 (0 - 0.05)	0.01 (0 - 0.03)	0.006 (0.001 - 0.01)	0.03 (0.01 - 0.1)	0.03 (0.006 - 0.1)
Uganda	2167.0 (1156.6 - 3905.5)	69.2 (39.7 - 113.4)	1705.2 (775.2 - 3341.9)	75.9 (25.4 - 144.3)	100.2 (35.4 - 243.5)	104.3 (0 - 237.7)	23.8 (0 - 62.0)	10.2 (0 - 29.1)	4.0 (0.2 - 11.8)	35.9 (10.9 - 62.4)	20.0 (4.5 - 37.1)
Zambia	1423.7 (849.0 - 2366.6)	276.6 (160.4 - 446.5)	811.3 (369.6 - 1582.6)	39.1 (13.1 - 74.4)	157.8 (56.2 - 382.2)	47.3 (0 - 107.1)	21.3 (0 - 47.5)	20.6 (0 - 48.4)	1.5 (0.3 - 3.3)	6.5 (1.4 - 12.9)	16.9 (4.7 - 29.7)
Zimbabwe	700.7 (439.9 - 1095.3)	227.1 (126.6 - 386.1)	307.5 (139.7 - 601.2)	16.8 (5.6 - 34.0)	75.2 (26.8 - 181.9)	26.2 (0 - 63.4)	9.9 (0 - 25.7)	10.6 (0 - 28.8)	0.5 (0.1 - 1.1)	2.9 (0.6 - 5.8)	4.6 (1.0 - 9.1)

References

1. PEPFAR, Monitoring, Evaluation, and Reporting Data for 2024, on file with the authors.
2. Stevens O, Sabin K, Anderson RL, Garcia SA, Willis K, Rao A, et al. Population size, HIV prevalence, and antiretroviral therapy coverage among key populations in sub-Saharan Africa: collation and synthesis of survey data, 2010–23. The Lancet Global Health. 2024;12(9):e1400-e12.
3. Jones HS, Anderson RL, Cust H, McClelland RS, Richardson BA, Thirumurthy H, et al. HIV incidence among women engaging in sex work in sub-Saharan Africa: a systematic review and meta-analysis. The Lancet Global Health. 2024;12(8):e1244-e60.
4. Nouaman MN, Becquet V, Plazy M, Coffie PA, Zebago C, Montoyo A, et al. Incidence of HIV infection and associated factors among female sex workers in Cote d'Ivoire, results of the ANRS 12361 PrEP-CI study using recent infection assays. PLoS One. 2022;17(11):e0271988.
5. McClelland RS, Richardson BA, Cherutich P, Mandaliya K, John-Stewart G, Miregwa B, et al. A 15-year study of the impact of community antiretroviral therapy coverage on HIV incidence in Kenyan female sex workers. AIDS. 2015;29(17):2279-86.
6. Lyons CE, Olawore O, Turpin G, Coly K, Ketende S, Liestman B, et al. Intersectional stigmas and HIV-related outcomes among a cohort of key populations enrolled in stigma mitigation interventions in Senegal. AIDS. 2020;34 Suppl 1(Suppl 1):S63-S71.
7. Kassanjee R, Welte A, Otwombe K, Jaffer M, Milovanovic M, Hlongwane K, et al. HIV incidence estimation among female sex workers in South Africa: a multiple methods analysis of cross-sectional survey data. Lancet HIV. 2022;9(11):e781-e90.
8. Faini D, Msafiri F, Munseri P, Bakari M, Lyamuya E, Sandstrom E, et al. The Prevalence, Incidence, and Risk Factors for HIV Among Female Sex Workers-A Cohort Being Prepared for a Phase IIb HIV Vaccine Trial in Dar es Salaam, Tanzania. J Acquir Immune Defic Syndr. 2022;91(5):439-48.
9. Kerrigan D, Mbwambo J, Likindikoki S, Davis W, Mantsios A, Beckham SW, et al. Project Shikamana: Community Empowerment-Based Combination HIV Prevention Significantly Impacts HIV Incidence and Care Continuum Outcomes Among Female Sex Workers in Iringa, Tanzania. J Acquir Immune Defic Syndr. 2019;82(2):141-8.
10. Kasamba I, Nash S, Shahmanesh M, Baisley K, Todd J, Kamacooko O, et al. Missed study visits and subsequent HIV incidence among women in a predominantly sex worker cohort attending a dedicated clinic service in Kampala, Uganda. JAIDS Journal of Acquired Immune Deficiency Syndromes. 2019;82(4):343-54.
11. Chabata ST, Hensen B, Chiyaka T, Mushati P, Musemburi S, Dirawo J, et al. The impact of the DREAMS partnership on HIV incidence among young women who sell sex in two Zimbabwean cities: results of a non-randomised study. BMJ Glob Health. 2021;6(4).
12. Jones HS, Hensen B, Musemburi S, Chinyanganya L, Takaruza A, Chabata ST, et al. Temporal trends in, and risk factors for, HIV seroconversion among female sex workers accessing Zimbabwe's national sex worker programme, 2009-19: a retrospective cohort analysis of routinely collected HIV testing data. Lancet HIV. 2023;10(7):e442-e52.
13. Hessou SPH, Glele-Ahanhanzo Y, Adekpedjou R, Ahoussinou C, Djade CD, Biaou A, et al. HIV incidence and risk contributing factors among men who have sex with men in Benin: A prospective cohort study. PLoS One. 2020;15(6):e0233624.
14. Stannah J, Soni N, Lam JKS, Giguère K, Mitchell KM, Kronfli N, et al. Trends in HIV testing, the treatment cascade, and HIV incidence among men who have sex with men in Africa: a systematic review and meta-analysis. The lancet HIV. 2023;10(8):e528-e42.
15. Dah TTE, Yaya I, Sagaon-Teyssier L, Coulibaly A, Kouame MJ, Agboyibor MK, et al. Adherence to quarterly HIV prevention services and its impact on HIV incidence in men who have sex with men in West Africa (CohMSM ANRS 12324 - Expertise France). BMC Public Health. 2021;21(1):972.
16. Graham SM, Okall DO, Mehta SD, Obondi E, Ng'ety G, Ochieng E, et al. Challenges with PrEP Uptake and Adherence Among Gay, Bisexual, and Other Men Who Have Sex with Men in Kisumu, Kenya. AIDS Behav. 2023;27(4):1234-47.
17. Kimani M, van der Elst EM, Chiro O, Oduor C, Wahome E, Kazungu W, et al. PrEP interest and HIV-1 incidence among MSM and transgender women in coastal Kenya. J Int AIDS Soc. 2019;22(6):e25323.
18. Sandfort TGM, Mbilizi Y, Sanders EJ, Guo X, Cummings V, Hamilton EL, et al. HIV incidence in a multinational cohort of men and transgender women who have sex with men in sub-Saharan Africa: Findings from HPTN 075. PLoS One. 2021;16(2):e0247195.
19. Wahome EW, Graham SM, Thiong'o AN, Mohamed K, Oduor T, Gichuru E, et al. PrEP uptake and adherence in relation to HIV-1 incidence among Kenyan men who have sex with men. EClinicalMedicine. 2020;26:100541.
20. Couderc C, Dembele Keita B, Anoma C, Wade AS, Coulibaly A, Ehouman S, et al. Is PrEP Needed for MSM in West Africa? HIV Incidence in a Prospective Multicountry Cohort. J Acquir Immune Defic Syndr. 2017;75(3):e80-e2.
21. Ramadhani HO, Crowell TA, Nowak RG, Ndembi N, Kayode BO, Kokogho A, et al. Association of age with healthcare needs and engagement among Nigerian men who have sex with men and transgender women: cross-sectional and longitudinal analyses from an observational cohort. J Int AIDS Soc. 2020;23 Suppl 6(Suppl 6):e25599.

22. Lippman SA, Lane T, Rabede O, Gilmore H, Chen YH, Mlotshwa N, et al. High Acceptability and Increased HIV-Testing Frequency After Introduction of HIV Self-Testing and Network Distribution Among South African MSM. J Acquir Immune Defic Syndr. 2018;77(3):279-87.
23. Sullivan PS, Phaswana-Mafuya N, Baral SD, Valencia R, Zahn R, Dominguez K, et al. HIV prevalence and incidence in a cohort of South African men and transgender women who have sex with men: the Sibanye Methods for Prevention Packages Programme (MP3) project. J Int AIDS Soc. 2020;23 Suppl 6(Suppl 6):e25591.
24. Artenie A, Stone J, Fraser H, Stewart D, Arum C, Lim AG, et al. Incidence of HIV and hepatitis C virus among people who inject drugs, and associations with age and sex or gender: a global systematic review and meta-analysis. Lancet Gastroenterol Hepatol. 2023;8(6):533-52.
25. Degenhardt L, Webb P, Colledge-Frisby S, Ireland J, Wheeler A, Ottaviano S, et al. Epidemiology of injecting drug use, prevalence of injecting-related harm, and exposure to behavioural and environmental risks among people who inject drugs: a systematic review. Lancet Glob Health. 2023;11(5):e659-e72.
26. Artenie A, Perry R, Mahaso M, Jankie T, McNaughton AL, Stone J, et al. HIV incidence and factors associated with HIV risk among people who inject drugs engaged with harm-reduction programmes in four provinces in South Africa: a retrospective cohort study. Lancet HIV. 2024;11(12):e823-e32.
27. United Nations Department of Economic and Social Affairs, Population Division. World Population Prospects 2024. https://population.un.org/wpp/. Accessed: 22nd May 2025.
28. https://aidsinfo.unaids.org. Accessed: 22nd May 2025.
29. Rosenberg NE, Shook-Sa BE, Liu M, Stranix-Chibanda L, Yotebieng M, Sam-Agudu NA, et al. Adult HIV-1 incidence across 15 high-burden countries in sub-Saharan Africa from 2015 to 2019: a pooled analysis of nationally representative data. The lancet HIV. 2023;10(3):e175-e85.
30. Government of Botswana: Botswana AIDS Impact Survey V 2021 (BAIS V): Report. National AIDS & Health Promotion Agency, Gaborone, Botswana. August 2023.
31. Population-Based HIV Impact Assessment https://phia-data.icap.columbia.edu.
32. Federal Ministry of Health, Nigeria. Nigeria HIV/AIDS Indicator and Impact Survey (NAIIS) 2018: Technical Report. Abuja, Nigeria. October 2019.
33. Simbayi L, Zuma K, Moyo S, Marinda E, Mabaso M, Ramlagan S, et al. South African national HIV prevalence, incidence, behaviour and communication survey, 2017. 2021.
34. Murchu EO, Marshall L, Teljeur C, Harrington P, Hayes C, Moran P, et al. Oral pre-exposure prophylaxis (PrEP) to prevent HIV: a systematic review and meta-analysis of clinical effectiveness, safety, adherence and risk compensation in all populations. BMJ open. 2022;12(5):e048478.
35. Eakle R, Gomez GB, Naicker N, Bothma R, Mbogua J, Cabrera Escobar MA, et al. HIV pre-exposure prophylaxis and early antiretroviral treatment among female sex workers in South Africa: results from a prospective observational demonstration project. PLoS medicine. 2017;14(11):e1002444.
36. Jana S, Ray P, Roy S, Kadam A, Gangakhedkar RR, Rewari B, et al. Successful integration of HIV pre-exposure prophylaxis into a community-based HIV prevention program for female sex workers in Kolkata, India. International journal of STD & AIDS. 2021;32(7):638-47.
37. Mboup A, Béhanzin L, Guédou FA, Geraldo N, Goma-Matsétsé E, Giguère K, et al. Early antiretroviral therapy and daily pre-exposure prophylaxis for HIV prevention among female sex workers in Cotonou, Benin: a prospective observational demonstration study. Journal of the International AIDS Society. 2018;21(11):e25208.
38. Reza-Paul S, Lazarus L, Maiya R, Haldar P, Rewari B, Venugopal M, et al. The Ashodaya PrEP project: lessons and implications for scaling up PrEP from a community-led demonstration project among female sex workers in Mysore, India. Global Public Health. 2020;15(6):889-904.
39. Sarr M, Gueye D, Mboup A, Diouf O, Bao MDB, Ndiaye AJ, et al. Uptake, retention, and outcomes in a demonstration project of pre-exposure prophylaxis among female sex workers in public health centers in Senegal. International journal of STD & AIDS. 2020;31(11):1063-72.
40. Chou R, Evans C, Hoverman A, Sun C, Dana T, Bougatsos C, et al. Preexposure Prophylaxis for the Prevention of HIV Infection: Evidence Report and Systematic Review for the US Preventive Services Task Force. JAMA. 2019;321(22):2214-30.
41. Stone J, Bothma R, Gomez GB, Eakle R, Mukandavire C, Subedar H, et al. Impact and cost-effectiveness of the national scale-up of HIV pre-exposure prophylaxis among female sex workers in South Africa: a modelling analysis. J Int AIDS Soc. 2023;26(2):e26063.
42. Choopanya K, Martin M, Suntharasamai P, Sangkum U, Mock PA, Leethochawalit M, et al. Antiretroviral prophylaxis for HIV infection in injecting drug users in Bangkok, Thailand (the Bangkok Tenofovir Study): a randomised, double-blind, placebo-controlled phase 3 trial. The Lancet. 2013;381(9883):2083-90.
43. Baeten JM, Donnell D, Ndase P, Mugo NR, Campbell JD, Wangisi J, et al. Antiretroviral prophylaxis for HIV prevention in heterosexual men and women. New England journal of medicine. 2012;367(5):399-410.
44. Thigpen MC, Kebaabetswe PM, Paxton LA, Smith DK, Rose CE, Segolodi TM, et al. Antiretroviral preexposure prophylaxis for heterosexual HIV transmission in Botswana. New England journal of medicine. 2012;367(5):423-34.

45. Marrazzo JM, Ramjee G, Richardson BA, Gomez K, Mgodi N, Nair G, et al. Tenofovir-based preexposure prophylaxis for HIV infection among African women. New England Journal of Medicine. 2015;372(6):509-18.
46. Peterson L, Taylor D, Roddy R, Belai G, Phillips P, Nanda K, et al. Tenofovir disoproxil fumarate for prevention of HIV infection in women: a phase 2, double-blind, randomized, placebo-controlled trial. PLoS clinical trials. 2007;2(5):e27.
47. Van Damme L, Corneli A, Ahmed K, Agot K, Lombaard J, Kapiga S, et al. Preexposure prophylaxis for HIV infection among African women. New England Journal of Medicine. 2012;367(5):411-22.
